# Supplementary material for: Safety, feasibility, acceptability and preliminary effects of the Neurofenix platform for Rehabilitation via HOMe Based gaming exercise for the Upper-limb post Stroke (RHOMBUS): results of a feasibility intervention study
Source: BMJ Open. 2022 Feb 27;12(2):e052555. doi: 10.1136/bmjopen-2021-052555 (PMC8886425; doi:10.1136/bmjopen-2021-052555)
Supplement: Author's manuscript [file bmjopen-2021-052555.draft_revisions.pdf]

BMJ Open is committed to open peer review. As part of this commitment we make the peer review history of every article we publish publicly available.

When an article is published we post the peer reviewers' comments and the authors' responses online. We also post the versions of the paper that were used during peer review. These are the versions that the peer review comments apply to.

The versions of the paper that follow are the versions that were submitted during the peer review process. They are not the versions of record or the final published versions. They should not be cited or distributed as the published version of this manuscript.

BMJ Open is an open access journal and the full, final, typeset and author-corrected version of record of the manuscript is available on our site with no access controls, subscription charges or pay-per-view fees (http://bmjopen.bmj.com).

If you have any questions on BMJ Open's open peer review process please email info.bmjopen@bmj.com

BMJ Open

Safety, feasibility, acceptability, and preliminary effects of the Neurofenix Platform for Rehabilitation via HOMe Based gaming exercise for the Upper-limb post-stroke (RHOMBUS): results of a feasibility intervention study.

Journal:	*BMJ Open*
Manuscript ID	bmjopen-2021-052555
Article Type:	Original research
Date Submitted by the Author:	19-Apr-2021

Complete List of Authors:	Kilbride, Cherry; Brunel University London, Department of Health Sciences<br>Scott, Daniel J M; Brunel University London, Department of Health Sciences; Neurofenix<br>Butcher, Tom; Brunel University College of Health and Life Sciences, Health Sciences<br>Norris, Meriel; Brunel University London, College of Health and Life Sciences<br>Warland, Alyson; Brunel University Department of Clinical Sciences, Clinical Sciences<br>Anokye, Nana; Brunel University London, Department of Health Sciences<br>Cassidy, Elizabeth ; Freelance academic and research supervisor<br>Baker, Karen; Brunel University London, Department of Health Sciences; Neurofenix<br>Athanasiou, Dimitrios; Neurofenix<br>Singla-Buxarrais, Guillem; Neurofenix<br>Nowicky, Alexander; Brunel University London, Department of Health Sciences<br>Ryan , Jennifer ; Royal College of Surgeons in Ireland, Public Health and Epidemiology

Keywords:	Stroke < NEUROLOGY, REHABILITATION MEDICINE, Adult neurology < NEUROLOGY

SCHOLARONE™
Manuscripts

I, the Submitting Author has the right to grant and does grant on behalf of all authors of the Work (as defined in the below author licence), an exclusive licence and/or a non-exclusive licence for contributions from authors who are: i) UK Crown employees; ii) where BMJ has agreed a CC-BY licence shall apply, and/or iii) in accordance with the terms applicable for US Federal Government officers or employees acting as part of their official duties; on a worldwide, perpetual, irrevocable, royalty-free basis to BMJ Publishing Group Ltd ("BMJ") its licensees and where the relevant Journal is co-owned by BMJ to the co-owners of the Journal, to publish the Work in this journal and any other BMJ products and to exploit all rights, as set out in our licence.

The Submitting Author accepts and understands that any supply made under these terms is made by BMJ to the Submitting Author unless you are acting as an employee on behalf of your employer or a postgraduate student of an affiliated institution which is paying any applicable article publishing charge ("APC") for Open Access articles. Where the Submitting Author wishes to make the Work available on an Open Access basis (and intends to pay the relevant APC), the terms of reuse of such Open Access shall be governed by a Creative Commons licence – details of these licences and which Creative Commons licence will apply to this Work are set out in our licence referred to above.

Other than as permitted in any relevant BMJ Author's Self Archiving Policies, I confirm this Work has not been accepted for publication elsewhere, is not being considered for publication elsewhere and does not duplicate material already published. I confirm all authors consent to publication of this Work and authorise the granting of this licence.

For peer review only

Safety, feasibility, acceptability, and preliminary effects of the Neurofenix Platform for **R**ehabilitation via **HOM**e **B**ased gaming exercise for the **Upper-limb** post-stroke (RHOMBUS): results of a feasibility intervention study.

Authors

Cherry Kilbride
Department of Health Sciences, Institute of Health, Medicine and Environment, Brunel University London, Uxbridge, UK

Daniel J M Scott
Department of Health Sciences, Institute of Health, Medicine and Environment, Brunel University London, Uxbridge, UK; Neurofenix, London, UK

Tom Butcher
Department of Health Sciences, Institute of Health, Medicine and Environment, Brunel University London, Uxbridge, UK

Meriel Norris
Department of Health Sciences, Institute of Health, Medicine and Environment, Brunel University London, Uxbridge, UK

Alyson Warland
Department of Health Sciences, Institute of Health, Medicine and Environment, Brunel University London, Uxbridge, UK

Nana Anokye
Department of Health Sciences, Institute of Health, Medicine and Environment, Brunel University London, Uxbridge, UK

Elizabeth Cassidy
Freelance Academic, London

Karen Baker
Department of Health Sciences, Institute of Health, Medicine and Environment, Brunel University London, Uxbridge, UK; Neurofenix, London, UK

Dimitrios A Athanasiou
Neurofenix, London, UK

Guillem Singla-Buxarrais
Neurofenix, London, UK

Alexander Nowicky
Department of Health Sciences, Institute of Health, Medicine and Environment, Brunel University London, Uxbridge, UK

Jennifer Ryan
Department of Public Health and Epidemiology, RCSI University of Medicine and Health Sciences, Dublin, Ireland; Department of Health Sciences, Institute of Health, Medicine and Environment, Brunel University London, Uxbridge, UK

Correspondence to:
Cherry Kilbride: cherry.kilbride@brunel.ac.uk
+44 (0)1895 268675
Brunel University London, Department of Health Sciences, Uxbridge UB8 3PH

Version 1

DATE: 18 April 2021

WORD COUNT (excluding title page, abstract, references, figures and tables): 3853

For peer review only - http://bmjopen.bmj.com/site/about/guidelines.xhtml

Abstract

Objectives

To investigate the safety, feasibility and acceptability of the Neurofenix platform for home-based rehabilitation of the upper-limb (UL).

Design

A non-randomised intervention design with a parallel process evaluation.

Setting

Participants' homes South-East England.

Participants

Thirty adults (≥18 years), minimum 12-weeks post-stroke, not receiving UL rehabilitation, scoring 9-25 on the Motricity Index (elbow and shoulder), with sufficient cognitive and communicative abilities to participate.

Interventions

Participants were trained to use the platform, followed by one-week of graded game-play exposure and six-weeks training, aiming for a minimum 45 minutes, 5 days/week.

Outcomes

Safety was determined by assessing pain and fatigue at 8 and 12-weeks, and adverse events (AEs). Intervention feasibility was determined by amount of specialist training and support required to complete the intervention, time and days spent training, and number of UL movements performed. Acceptability was assessed by a satisfaction questionnaire and semi-structured interviews.

Results

Participants [14 women; mean (SD) age 60.0 (11.3) years] were a median of 4.9 years post-stroke (minimum-maximum: 1-28 years). Twenty-seven participants completed the intervention. The odds of having shoulder pain were lower at 8-weeks (OR 0.45, 95% CI 0.24 to 0.83, p=0.010) and 12-weeks (OR 0.46, 95% CI 0.25 to 0.86, p=0.014) compared to baseline. There was no change in fatigue. Thirty mild and short-term AEs and 1 serious (unrelated) AE were reported by a total of 19 participants. Median time for training, over 1-3 sessions, was 98 minutes per participant (minimum-maximum 70-290 minutes). Participants trained with the platform for a median of 17.4 hours over 7-weeks (minimum-maximum: 0.3-46.9 hours), equating to a median of 149 minutes per week. The median satisfaction score was 36 out of 40.

For peer review only - http://bmjopen.bmj.com/site/about/guidelines.xhtml

Conclusion

The Neurofenix platform is a safe, feasible and acceptable way to support UL training for people at least three months post-stroke.

Ethics approval

Ethics approval granted from Brunel University London (10249-MHR-Mar/2018-12322-2).

Trial registration number

ISRCTN60291412 (doi 10.1186/ISRCTN60291412). Protocol version:1.0; pre-results.

Funding

Innovate UK.

For the BMJ Open Submission website (ScholarOne)

Keywords

Rehabilitation, stroke, upper-limb, virtual reality, gaming, protocol, feasibility.

Keywords (MeSH)

Stroke therapy/rehabilitation/methods

Upper extremity/limb

Virtual reality stroke rehabilitation

Video games

Feasibility

For peer review only

Article Summary

Strengths and limitations of this study

1. A feasibility trial developed following Medical Research Council Framework for the development of complex interventions.
2. Mixed-method approach for answering feasibility and acceptability objectives.
3. Fidelity activity data were objectively measured through the platform.
4. Inclusive of people with higher levels of UL impairment.
5. No control group or blinded assessor.

For peer review only

For peer review only - http://bmjopen.bmj.com/site/about/guidelines.xhtml

Introduction

Upper-limb (UL) dysfunction is a common and major contributor to physical disability following stroke.[1] Approximately two-thirds of stroke survivors experience upper limb impairment [2] and only 5% to 20% achieve full UL recovery at six months post-stroke.[3]

High intensity UL stroke rehabilitation improves upper limb recovery with a positive dose-response relationship for individuals in the acute and chronic phases after stroke.[4] UL training provided in rehabilitation settings typically falls below the hundreds of repetitions considered necessary to drive functional recovery.[5,6]

Virtual reality (VR) platforms using interactive computer-based games have emerged as a feasible and motivating way of supporting UL rehabilitation at intensities significantly higher than conventional therapy.[7,8] However, bespoke VR devices are often unwieldly, complex, and expensive and while commercially available devices may be more accessible, they often demand sophisticated movement combinations which are not possible for many stroke survivors to perform.[9-11]

The Neurofenix platform is a novel, affordable and portable VR device specifically developed by stroke survivors, specialist neuro-physiotherapists and bioengineers for gamification of post-stroke UL rehabilitation (www.neurofenix.com). The platform enables and encourages stroke survivors to exercise their arm and hand at home with little or no assistance. The overall aim of this study was to investigate the safety, feasibility and acceptability of the platform for home-based rehabilitation of the UL. Additional objectives were (1) to assess the feasibility of conducting a definitive trial of the clinical and cost-effectiveness of the intervention, (2) to understand factors relating to people with stroke and the intervention that may impact on fidelity to the intervention, and (3) to examine preliminary effects of the intervention.

Methods

The study is reported in accordance with guidance on reporting pilot and feasibility studies.[12] Further details on trial procedures are detailed in the protocol.[13]

Patient and Public Involvement

Stroke survivors were involved in the iterative development of the Neurofenix platform. Two further stroke survivors acted in an advisory capacity during the study, providing input to the protocol and trial documentation, and dissemination.

For peer review only - http://bmjopen.bmj.com/site/about/guidelines.xhtml

Trial Design

The RHOMBUS study used a non-randomised intervention design with a parallel process evaluation. Participants were familiarised, set-up and trained to use the device by a research therapist, followed by one-week of graded exposure to game-play, and subsequent six-week independent training phase. The device was removed at the end of the 7-week training period. Assessments were performed at baseline, 8, and 12-weeks.

Participants

Participants were recruited from a university stroke database, via gatekeepers for three community stroke groups and the ISRCTN Registry website between April and September 2018. Inclusion criteria were: 1) aged 18 years or over; 2) capacity to consent; 3) self-reported diagnosis of stroke; 4) 12-weeks minimum post-stroke and not in receipt of UL rehabilitation; 5) mild-to-severe reduction in arm function post-stroke (Motricity Index (MI) score between 9-25 for elbow and shoulder movement); 6) able to sit or stand independently, with or without an aid, for a minimum of 5 minutes; and 7) sufficient English to participate in the intervention and assessments. Exclusion criteria were: 1) unstable medical conditions; 2) uncontrolled photosensitive epilepsy; 3) acquired brain injury from other causes, bilateral or cerebellar lesions; 4) uncompensated visual neglect, hemianopia or uncorrected visual field deficits; and 5) pre-existing, unremitting arm pain at rest.

Intervention

The intervention is described in detail in the protocol.[13] Briefly, the Neurofenix platform is a digital device consisting of non-immersive VR software in the form of an App on a tablet and Bluetooth-connected hand controller (NeuroBall) and arm controllers (Neurobands). The NeuroBall is secured to the impaired hand through a system of straps and elastic finger holds. Motion sensors within the NeuroBall detect UL movements and translate these to control games displayed on a tablet. For those without sufficient strength and/or range of movement in the hand and wrist to control the NeuroBall, the NeuroBands are used as an alternative. The NeuroBands are two small motion sensors; the first straps around the upper arm and the second around either the forearm or the hand. If the second NeuroBand is placed on the forearm, then elbow flexion and extension can be used to control the games, and if placed around the hand, wrist flexion and extension can be used. Participants can choose from seven games specifically developed for UL rehabilitation (see www.neurofenix.com).

For peer review only - http://bmjopen.bmj.com/site/about/guidelines.xhtml

Two research therapists attended two half-day training sessions on technical and operational procedures relating to the device. Neurofenix engineers provided additional technical support to the therapists and participants throughout the study if required.

Following the baseline assessment, a research therapist attended the participant's home. The visit commenced with a brief questionnaire about the participant's prior experience with technology and video games and confidence in using new technology. The pace and depth of subsequent teaching was based on the participant's experience and confidence. The participant was then given the Neurofenix platform consisting of a NeuroBall or two NeuroBands, a tablet, a tablet stand, chargers, a handbook, and a one-page "Quickstart" guide.

A research therapist trained the participant in how to use the platform independently or with the help of a carer if requested. Specifically, participants were taught safety precautions and were trained to (1) turn the device and tablet on and off, (2) don and doff the device, (3) calibrate the device, (4) play each of the games on the tablet, (5) navigate the menus, (6) track their progress, (7) use the handbook and (8) charge the devices. The research therapist advised each participant on the starting duration and frequency of use based on signs of pain and fatigue during the visit. Participants were advised to gradually increase the amount of use over the first week, with the aim of achieving a minimum of 45 minutes of training on 5 days a week by the end of the week, and to continue with this duration and frequency for the subsequent 6-weeks, increasing the dose if able. Participants independently chose which games to play during the intervention. Participants received two scheduled phone calls from the research therapists during week -1 and week-3 to ensure no clinical problems or technical faults had occurred. After this second call, participants had no input from the research team, but were advised to contact the team if they needed clinical or technical support. Participants were contacted at the end of 7-weeks, asked to stop using the device, and arranged a time for the therapist to collect the device.

Procedures

Two research therapists completed assessments in participants' homes. Socioeconomic characteristics, stroke-specific characteristics and global disability were measured using the simplified modified Rankin Scale questionnaire (smQRs); higher score represents greater disability (range 1-5).[14,15]

Outcomes

Arm and hand function were assessed objectively using the Action Research Arm Test (ARAT), higher scores indicate better function.[16-18] UL impairment was assessed using the Fugl-Meyer

For peer review only - http://bmjopen.bmj.com/site/about/guidelines.xhtml

Assessment–upper-limb (FMA–UE); higher scores indicate less impairment.[19,20] Participation was measured on the 10-item Subjective Index of Physical and Social Outcome (SIPSO); higher scores signify increased ability to reintegrate to a 'normal' lifestyle.[21] Self-reported arm use during functional tasks was measured using the 28-item Motor Activity Log (MAL); higher scores indicate more activity.[22,23]

The Modified Modified Ashworth Scale (MMAS) was used to assess spasticity of the shoulder adductors and internal rotators, elbow, wrist and finger flexors.[24] The highest spasticity score within the UL was selected for analysis. Passive range of movement (PROM) of the shoulder (flexion, abduction, external rotation), elbow (flexion and extension) and wrist (extension) were assessed using goniometry.[25] Quality of life was assessed using the EQ-5D-5L questionnaire.[26] A utility value was calculated using the cross-walk function;[27] a higher value indicates better quality of life. A modified version of the Client Service Receipt Inventory (CSRI) was used to assess health service use.[28,29]

Safety

Safety was determined by assessing pain, fatigue and adverse events (AEs). At 8- and 12-weeks, participants were asked if they experienced shoulder pain over the past 7 days, and if so to rate the intensity of shoulder pain on a 10-point visual analogue scale (VAS), with higher scores indicating more pain. Fatigue was assessed using the Fatigue Severity Scale (FSS-7).[30] At the 8- and 12-week assessment, participants were asked if they had experienced an AE since their last contact with the research team using standardised questioning. An AE is any untoward medical occurrence affecting a participant during the study which may or may not be related to the study intervention. A serious AEs (SAEs) is an AE that results in death, is life threatening, requires hospitalisation or results in a disability or incapacity.

Feasibility

Feasibility of the intervention was determined by recording the number of people who received the training sessions, the number and duration of sessions required, and the number and duration of clinical or technical phone calls and home visits required. Post-training confidence with the Neurofenix Platform was assessed with a 10-point VAS (higher score equates to more confidence).

Feasibility was also determined by assessing fidelity to the intervention. Fidelity was assessed as time spent training the UL with the platform, number of days training, and number of UL movements performed. Sensors were used to automatically collect data from the Neurofenix

For peer review only - http://bmjopen.bmj.com/site/about/guidelines.xhtml

platform, which was stored on the participant's tablet until remotely downloaded by a member of the research team.

Acceptability

The Quebec User Evaluation of Satisfaction with Assistive Technology (QUEST 2.0) [31] was completed at 8-weeks to assess user satisfaction of the device; higher scores indicate higher satisfaction. Semi-structured interviews were completed with a purposive sample (male, female, high and low user engagement) of 18 participants to explore the acceptability of the intervention, and the experience of taking part in the study. Interviews were conducted by a research therapist (typically who did not provide training to that participant) and the analysis was undertaken by a member of the research team who did not otherwise have contact with participants (EC). Carers or spouses involved in the intervention were also invited to take part as a dyad.

Feasibility of a definitive trial

The feasibility of conducting a definitive trial was assessed by examining recruitment and retention rates, outcome measure completion and reasons for missing data associated with the data collection tools.

Sample Size

Samples sizes between 24 and 50 are recommended for feasibility studies.[32,33] We estimated 30 individuals would be sufficient to address the aims of this study.

Data analysis

Distribution of data was explored using Q-Q plots, histograms and cross-tabulations. Descriptive statistics (e.g., mean, standard deviation, median, interquartile range) were used to report data. Generalised estimating equations with an exchangeable working matrix and robust standard errors were used to examine change between baseline and follow-up assessments on the VAS, FSS, ARAT, FMA-UE, MMAS, MAL-28 (amount of use score only), SIPSO and goniometry. A generalised estimating equation with a logit link, exchangeable working matrix and robust standard errors was used to examine if the odds of shoulder pain changed over time.

Associations between total time spent exercising the UL with the platform, number of repetitions performed and age, ARAT score at baseline, FMA-UE score at baseline, confidence with new technology at baseline, post-training confidence with the Neurofenix platform were examined using Spearman's correlations. Associations between total time spent exercising the UL with the platform, number of repetitions performed, and gender were examined using Mann Whitney U tests.

For peer review only - http://bmjopen.bmj.com/site/about/guidelines.xhtml

Table 1. Baseline characteristics of RHOMBUS participants

	n (%)	Mean (SD)	Median (IQR),	Min-max
Age, year	30	60 (11.3)	60 (12),	36 to 85
Women	14 (47)	-	-	
**Ethnicity**				
White	15 (50)	-	-	
Asian	12 (40)	-	-	
Black	2 (7)	-	-	
Mixed	1 (3)			
Stroke chronicity, year	30	5.5 (5.4)	4.9 (4.5)	1 to 28
Stroke type (self-report)				
Haemorrhagic	17 (57)			
Ischaemic	12 (40)			
Not known	1 (3)			
**smRSq**				
1.0	3 (10)			

Qualitative data from the semi-structured interviews were analysed using the five stage Framework Analysis method.[34] Framework Analysis incorporates both deductive and inductive coding and enabled trial processes and experiences to be explored and reported. This method provides a strong audit trail of the analytical process, which enhances transparency.[35] One person (EC) independent to the delivery team led the analysis. Four researchers (EC, MN, TB, DS) independently coded the same three transcripts, then discussed and agreed codes. A coding framework was then developed and used to code all the interviews.

Results

Thirty participants were recruited to the study between April and July 2018 (Figure 1). Participants (14 women) were mean (SD) 60.0 (11.3) years (minimum-maximum: 36-85 years), and a median of 4.9 years post-stroke (minimum-maximum: 1-28 years) (Table 1). The majority scored 2 or 3 on the smRSq. Mean (SD) FMA-UE score was 33 (16.4) and median (IQR) ARAT score was 8 (20).

Insert Figure 1 near here.

For peer review only - http://bmjopen.bmj.com/site/about/guidelines.xhtml

2.0	11 (36)			
3.0	13 (43)			
4.0	1 (3)			
5.0	2 (6)			
FMA-UE (0-66)	30	33 (16.4)	33 (31),	8 to 63
ARAT (0-57)	30	16 (17.5)	8 (20),	0 to 57
MMAS worse score (0-5)	30	2 (0.98)	2 (2),	0 to 3
NIHSS	30	4.1 (3.1)	3 (3),	1 to 14
Played computer games before stroke (Yes)	19 (63)	-	-	
Played computer games since stroke (Yes)	15 (50)	-	-	
Owned a tablet, computer, console or smartphone (Yes)	28 (93)	-	-	
Confidence pre training with new technology (0-10)	30	5.63 (2.7)	5 (4)	
Confidence with NeuroPlatform post training (0-10)	29*	7.6 (2.24)	8 (3)	

*One participant withdrew during training. SD: standard deviation; IQR: inter quartile range; smRSq: simplified modified Rankin scale questionnaire; FMA-UE: Fugl Meyer upper extremity; ARAT: action research arm test; MMAS: modified modified Ashworth scale; NIHSS: national institute of health stroke scale.

For peer review only

Safety

The odds of having shoulder pain were lower at 8-weeks (OR 0.45, 95% CI 0.24 to 0.83, p=0.010) and 12-weeks (OR 0.46, 95% CI 0.25 to 0.86, p=0.014) compared to baseline. Mean intensity of shoulder pain, as assessed on a VAS, was lower at 12-weeks compared to baseline (mean difference -0.69, 95% CI -1.37 to 0.004, p=0.052; Table 2). There was no change in FSS-7 score, at 8 or 12-weeks in comparison to baseline (Table 2). Thirty AEs and 1 SAE were reported by a total of 19 participants (Table 3). Nineteen AEs were deemed probably or possibly related to the intervention; the AEs were mild and short-term and no participants withdrew due to an AE. The SAE was unrelated.

Feasibility

Thirty participants completed the training session (Table 4). Twenty-five participants needed one training session, three needed two sessions, and two needed a third session. The median time for training, over 1-3 sessions, was 98 minutes per participant (minimum-maximum 70-290 minutes). Median (IQR) participant confidence with the device after the initial training session was 8 (2) out of 10. Nine participants (30%) contacted the research team for technical support during the intervention period. Five participants (17%) contacted the research team for clinical support. The total number of visits, calls and their median durations are reported in Table 5.

Fidelity to the intervention

Participants trained with the Neurofenix platform for a median (minimum-maximum) of 17.4 (0.3-46.9) hours over 7-weeks, equating to a median of 149 minutes per week. Participants trained for a mean (SD) of 25.4 (15.9) days over 7-weeks (minimum-maximum: 1-48 days), equating to mean (SD) 3.6 (2.3) days per week. Eight participants (26.7%) achieved at least 225 minutes per week during the study, which equates to the recommended 45 minutes of training per day on five days per week. Participants performed a median (minimum-maximum) of 15,092 (222-43,999) UL movements over 7-weeks.

No association was found between time spent training over 7-weeks and age (rho=0.24, p=0.203), ARAT (rho=0.06, p=0.744), FMA-UE scores (rho=0.003, p=0.987), confidence with new technology (rho=0.13, p = 0.505), confidence post-training (rho=-0.05, p=0.784). There was also no difference in time spent training between men and women (p=0.061). No association was found between number of UL movements over 7-weeks and age (rho=0.18, p=0.351), ARAT (rho=0.10, p=0.614), FMA-UL (rho=0.06, p=0.761), confidence with new technology (rho=0.15, p=0.427), confidence post-training (rho=-0.005, p=0.979). There was also no difference in number of UL movements between men and women (p=0.105).

For peer review only - http://bmjopen.bmj.com/site/about/guidelines.xhtml

For peer review only

Acceptability

The median score on the Quebec User Evaluation of Satisfaction with assistive Technology (QUEST) was 36 out of 40 (range 21 to 40). The device was well received and provided motivating UL training (Box 1). Motivating factors included enjoyable games, positive in-game feedback, competition, and challenge, engagement with more purposeful games than prescribed home exercises. Participants suggested that a greater variety of games and more challenging levels might enhance engagement with the platform. The home-based self-directed training provided by the device and its compact size was positively appraised because it offered a structured practice schedule and set amount of time to train and enabled practice to be planned around daily life.

Preliminary effects

The mean difference in outcomes between baseline and 8 and 12-weeks respectively, is reported in Table 6. Scores on the FMA-UE (mean difference 2.68, 95% CI 0.86 to 4.49; p=0.004) and MAL (mean difference 0.19, 95% CI 0.06 to 0.32; p=0.004) improved between baseline and 8-weeks. SIPSO total score increased between baseline and 12-weeks (mean difference 2.17, 95% CI 0.32 to 4.01; p=0.021). Passive range of shoulder external rotation, elbow flexion and wrist extension increased between baseline and 8-weeks, and between baseline and 12-weeks (p<0.05; Table 6). No changes were found for other outcomes.

Feasibility of conducting a definitive trial

Fifty people expressed interest in participating in the study. Twenty were excluded for reasons outlined in Figure 1, resulting in 60% being recruited. Three people withdrew from the intervention; reasons for withdrawing are shown in Figure 1. All participants completed all outcome measures at baseline. Of the three people who withdrew from the intervention, two withdrew from the study and did not complete assessments at 8 or 12 weeks. One additional person did not complete the 8-week and 12-week assessment, respectively, resulting in 90% retention at follow-up assessments. In addition to the 10% missing data due to loss to follow-up, the FSS-7 was not assessed at 8-weeks, and shoulder external rotation was not assessed for one person at 12-weeks due to human error, and one person refused to complete the ARAT at 12-weeks. Data about medication dosage in the modified CSRI was missing for four participants.

For peer review only - http://bmjopen.bmj.com/site/about/guidelines.xhtml

Table 2. Change in scores on 7 Item Fatigue Severity Scale (FFS-7) and Visual Analogue Scale for pain (VAS) between baseline and 8, and baseline and 12-weeks.

	**Baseline (n=30)**	**8-weeks (n=27)**	**Change; 8-weeks – baseline (n=27)**	**12-weeks (n=27)**	**Change; 12-weeks – baseline (n=27)**
	**Mean (SD)**	**Mean (SD)**	**Mean difference (95% CI); p value**	**Mean (SD)**	**Mean difference (95% CI); p value**
Fatigue FSS-7[a] (7-49)	28.5 (13.2)	25.8 (13.9)	-1.59 (-6.51 to 3.34); 0.528	26.1 (12.5)	-1.98 (-5.75 to 1.79); 0.304
VAS-pain (0-10)	2 (2.24)	1.7 (2.10)	-0.10 (-.73 to 0.53); 0.756	1.22 (1.60)	-0.69 (-1.37 to 0.004); 0.052

[a]n=26 for 8-weeks

For peer review only - http://bmjopen.bmj.com/site/about/guidelines.xhtml

Table 3. Adverse events and serious adverse events reported during study

Event Type	Number of events	Attribution			People reporting event, (n)
		Number of probably related events	Number of possibly related events	Number of unrelated events	
SAE	1	0	0	1	1
AE	30	17[a]	2[b]	11[c]	19

[a]muscle fatigue/soreness/discomfort (n=9); eye strain (n=2); upper-limb pain (n=4), self-reported increase in spasticity (n=2)
[b]neck pain (n=2)
[c]fatigue (n=2); falls (n=4); blurred vision (n=1); headache (n=4)

For peer review only - http://bmjopen.bmj.com/site/about/guidelines.xhtml

Table 4. Training sessions required per participants and duration of training

	Number of participants receiving, n (%)	Duration, minutes		
		Mean (SD)	Median (IQR)	Minimum-maximum
**Training sessions**				
First training session	30 (100)	95.23 (19.5)	92 (25)	55-150
Second training session	3 (10)	48.33 (35.5)	55 (70)	10-80
Third training session	2 (6.7)	95.0 (7.1)	95 (10)	90-100

IQR: interquartile range; SD: standard deviation

Table 5. Total number of phone calls and home visits (technical and clinical)

	Number of calls/visits	Duration, minutes per call/visit		
		Mean (SD)	Median (IQR)	Minimum-maximum
**Clinical**				
Phone calls	10	12.0 (6.4)	10 (9)	4-19
Home visits	5	24.0 (8.2)	30 (15)	15-30
**Technical**				
Phone calls	17	13.0 (10.3)	13 (10)	2-36
Home visits	15	49.0 (41.3)	35 (62.5)	5-123

IQR: interquartile range; SD: standard deviation

For peer review only

Participant number and pseudonym	Quotation (page and line numbers)
	**Training and Support**
P16 Ann (moderately impaired upper-limb)	*'He went through everything. He went through all the games as well, which was good, so that helped. So, it just gives you confidence and reassurance in what you're doing.' (7, 266-268).*
P22 Bal (moderately impaired upper-limb):	*'If I didn't understand or if the response wasn't what I was expecting then I would refer to the detailed instructions to see if I could improve (5, 192-94). Very easy [to use] […] The instructions were very clear (6, 229-237). [...] it's always useful to fall back on if you, if you can't cope.' (6, 247-48).*
P19 Sam (moderately impaired upper-limb):	*'Like I was stuck on a game, so I used the quick start just to find out if I was doing something wrong.' (13, 553-54).*
P28 Terry's wife Fran	*'I thought the quick start guide on that sheet was very helpful 'cos I'd often look at that while we were setting you up, weren't... didn't I, Terry, and that was very helpful, but then we didn't need it as time went on.'*
	**Acceptability**
P1 Iris (moderately impaired upper-limb)	*' I was trying to get to level 20 […] Yeah, I was getting into it […] I enjoyed the game because every time... If you didn't get to a, a certain point it will always say, 'Re-try' or 'Re-play' or 'Start again', and I like that (17, 777-94). And the message after was, was to tell you how many minutes you played for and 'Congratulations, see you tomorrow, bye', and that's it, I like that. [...] It was like you had a friend talking to you.' (21, 979-80).*
P6 Mark (mildly impaired upper-limb):	*'It was a little hard at first, right, because I used to be pressing it and pressing it but, er, anyth... anyway I got used to it like.' (21, 956-57).*
P16 Ann (moderately impaired upper-limb)	*'I like the ones that I can, that I actually can achieve, yeah. It's no good... I don't like ones that I know that I'm never gonna get to or never gonna do, I like ones that I can... Yeah, the easier ones for me that I can achieve relatively easy. Obviously it's gotta push you a little bit but not too much.' (14, 648-51)*

For peer review only - http://bmjopen.bmj.com/site/about/guidelines.xhtml

P22 Bal (moderately impaired upper-limb)	*'I have found it, er, quite entertaining but sometimes, because, er, there's not much variety, it's the same music, the same games, it's a bit boring to do it every day. […] If there was a bit more variety or slightly different games, or even the games you could adjust so that, er, it was, er, slightly different, then I think it might not be so boring.' (15, 650-58).*

Box 1. Illustrative quotations from the qualitative interviews

For peer review only

For peer review only - http://bmjopen.bmj.com/site/about/guidelines.xhtml

Table 6 Change in outcomes between baseline and 8-weeks and baseline and 12-weeks.					
**Outcome**	**Baseline (n=30)**	**8-weeks (n=27)**	**Change; 8-weeks - baseline (n=27)**	**12-weeks (n=27)**	**Change; 12-weeks - baseline (n=27)**
	**Mean (SD)**	**Mean (SD)**	**Mean difference (95% CI); p value**	**Mean (SD)**	**Mean difference (95% CI); p value**
ARAT[a] (0-57)	15.9 (17.5)	16.2 (17.3)	0.17 (-0.86 to 1.21); 0.743	18.2 (19.1)	0.63 (-0.97 to 2.23); 0.440
FMA-UE (0-66)	32.6 (16.4)	35.2 (17.6)	2.68 (0.86 to 4.49); 0.004	34.4 (17.8)	1.49 (-0.38 to 3.37); 0.118
SIPSO; total score (0-40)	21.9 (9.3)	23.5 (9.3)	1.18 (-0.81 to 3.18); 0.245	24.3 (8.1)	2.17 (0.32 to 4.01); 0.021
MAL (0-5)	0.89 (1.19)	1.08 (1.16)	0.19 (0.06 to 0.32); 0.004	1.10 (1.19)	0.13 (-0.02 to 0.28); 0.080
MMAS (0-5)	2.0 (1.0)	1.8 (1.0)	-0.16 (-0.38 to 0.06); 0.157	2.1 (1.1)	0.08 (-0.19 to 0.35); 0.560
EQ-5D-5L; utility score	0.49 (0.32)	0.56 (0.30)	0.07 (-0.009 to 0.142); 0.088	0.54 (0.32)	0.44 (-0.02 to 0.10); 0.145
Shoulder flexion[b]	121.3 (18.2)	123.8 (19.4)	3.09 (-2.17 to 8.37); 0.249	119.9 (21.2)	-0.26 (-7.31 to 6.77); 0.940
Shoulder external rotation[ab]	34.0 (19.9)	41.5 (16.7)	7.10 (2.36 to 11.84); 0.003	40.6 (16.4)	6.52 (0.02 to 13.03); 0.049
Shoulder abduction[b]	107.6 (17.0)	111.1 (19.3)	3.21 (-2.00 to 8.42); 0.228	108.7 (19.7)	1.48 (-4.66 to 7.63); 0.636
Elbow flexion[b]	142.3 (8.7)	145.9 (5.6)	3.28 (0.44 to 6.12); 0.024	148.4 (6.3)	6.00 (3.20 to 8.80); <0.001
Elbow extension[b]	-4.26 (10.84)	-4.14 (12.06)	0.54 (-1.94 to 3.03); 0.669	-4.29 (11.00)	0.39 (-1.79 to 2.58); 0.724
Wrist extension[b]	58.5 (29.1)	67.1 (19.6)	10.18 (1.31 to 19.05); 0.024	67.81 (23.22)	10.9 (1.8 to 20.1); 0.019

[a]n=26 at 12-weeks
[b] Passive range of movement, degrees

For peer review only - http://bmjopen.bmj.com/site/about/guidelines.xhtml

Discussion

The results of this feasibility study indicate that the Neurofenix platform is a safe, feasible and acceptable intervention for UL rehabilitation for participants more than one-year post-stroke with mild-to-severe arm impairment. Participants experienced a reduction in shoulder pain over time, no change in fatigue, and mild AEs. On average, participants trained with the Neurofenix platform four days per week, for 149 minutes per week. Even amongst this group with relatively high UL impairment, 27% achieved at least 225 minutes per week during the study, which equates to the recommended 45 minutes of training per day on five days per week. Participants were highly satisfied with the rehabilitation programme (see Box 1). Additionally, loss to follow-up and missing data were minimal, which in combination with data on safety, feasibility and acceptability, suggest that a definitive RCT is feasible.

Our findings on safety are consistent with similar studies.[36-38] Self-limited use of the device permitted self-management of the risk of muscle fatigue and may explain the improvement in pain. We systematically assessed AEs, regardless of their relatedness to the intervention, which may explain the relatively high proportion of participants who experienced at least one event. However, AEs were mild, and the majority were unrelated to the intervention, as found in comparable studies.[36,37]

Recruitment targets were achieved within the specified timeframe of five months; 60% of those screened were enrolled in the trial. The eligibility to enrolment ratio compares favourably to a multicentre study that examined the use of the Nintendo Wii for UL stroke rehabilitation in the UK where a low enrolment figure (4%) was achieved using broad eligibility screening in ten stroke centres.[37] Our strategy of using three recruitment streams, targeting local stroke groups and newsletters for stroke survivors, positively influenced engagement with the study and enrolment. However, other modes of recruitment may be necessary to enrol sufficient numbers for a definitive trial. Attrition was within acceptable limits (<20%),[39] and, compared to similar trials, consistent with figures reported by Adie et al [37] and lower than those reported by Standen et al.[40] Both studies provided comparable levels of support and participants were of similar age to those in our study. There are no obvious differences to account for the increased drop out in the Standen et al trial.[40]

Outcome data reporting was very good. Some participants found the assessments time consuming and fatiguing. We assessed the four core measures recommended for stroke trials.[41] We also included safety measures for pain and fatigue, and the SIPSO because there is no consensus about the most

For peer review only - http://bmjopen.bmj.com/site/about/guidelines.xhtml

appropriate measure of participation in stroke trials.[41] To balance the need to fully test our research questions against the need to use outcome measures judiciously [41] we additionally measured spasticity, passive range of movement and the MAL.

Most participants in this trial required one training session to confidently use the device and no further in-trial technical or clinical support. These results compare favourably with Standen et al [40] suggesting the training and follow-up support required to implement this intervention is feasible in clinical practice. A minority of participants required phone calls and home visits to help with clinical and technical issues. These problems may impede fidelity and, as previously reported,[40] are potential barriers to independent home use of rehabilitation devices. The Neurofenix platform was designed to enable independent home-based rehabilitation. This study showed that although technology was effectively leveraged to reduce clinical contact time, individually paced home-based instruction and support services were necessary to effectively deliver the intervention. These additional hours would need to be factored into a fully scaled trial to ensure that technological interventions are not studied as a dose matched control but on how they would be rolled out and used by service users and clinicians in real life. A future RCT should examine the cost-effectiveness of the intervention compared to usual care, given the minimal therapist time involved in providing this intervention in a community setting.

Just over a quarter of participants met the target of training for 45 minutes per day on a 5 day a week training cycle. However, consistent with previous studies,[37,40] most participants did not meet training targets and the duration of use and number of movement repetitions varied widely across participants. Standen at al [40] reported that family support, where available, was crucial to successful game-play. We also found that participants with severe UL impairment were dependent on carers for set-up. Participants starting from a low baseline may apply an equivalent effort as those with less impairment without meeting training targets which suggests that personalised training goals may be useful.

Consistent with our findings, previous studies reported that participants generally enjoy using VR rehabilitation devices.[36,42,43] The compact size and light weight of the devices and the tablet facilitated the use of the platform in the home environment and positively influenced satisfaction and acceptability. Qualitative data suggested that a broader selection of games with a wide range of difficulty levels and the introduction of real time in-play competition and comparison may increase motivation and encourage persistence. These findings are consistent with game theory for stroke

For peer review only - http://bmjopen.bmj.com/site/about/guidelines.xhtml

rehabilitation which emphasises the importance of in-built skill level adaptation, feedback, competition, and socialisation.[44] Adaptations to the games may be required prior to conducting a definitive trial to increase motivation.

To be maximally inclusive, stroke survivors with moderate to severe arm impairment were included in the study which may be considered a strength. Participants were relatively young compared to the UK average age of onset for stroke [45] but with a comparable age distribution to participants included in similar studies.[36,40,46] Older people may be thought to be less inclined to use rehabilitation technology, but we found no associations between age and time spent training using the device. The Neurofenix platform was custom designed for therapeutic use, which confers advantages over commercial gaming devices.[44] The device calibrates individual movement repetitions but key design features that further augment motivation and persistence such as personalisation through in-game adaptation [44,47] were not included in our device at the time of the study.

Conclusion

The Neurofenix platform is a safe, feasible, and acceptable means of delivering upper-limb rehabilitation to people following stroke. Iterative design continues to develop and improve the platform by incorporating game theory to encourage motivation and persistence. As the effects of stroke vary considerably across individuals, and as individuals are likely to have different technical capacities, a supported remote training model such as that used in this trial is likely to be needed in a definitive trial.

For peer review only

Author contributions

CK and GSB conceived the study, DS, TB, AN, JR, NA, AW, MN designed the study. GSB, DA, DS, TB, and CK designed the intervention. CK led the running of the trial. DS and TB led the collection, management, and analysis of the data. KB led blinded ARAT assessment. MN led the process evaluation. JR led the statistical analysis. NA led the evaluation of the feasibility of the economic evaluation. EC led the qualitative analysis. All authors have read and approved the final manuscript.

Study sponsor and funders were not involved in study design; collection; management; analysis and interpretation of the data; writing of the protocol; and the decision to submit the report for publication.

Acknowledgements

The authors would like to thank two stroke survivors who assisted the development of the intervention and advised on the protocol, trial documentation and dissemination. Further thanks to the group facilitators of Different Strokes and the Action for Rehabilitation from Neurological Injury. Thanks to Professor Christina Victor for her support.

Funding statement

This work was supported by Innovate UK grant number 104188, (the views expressed are those of the authors and not necessarily those of Innovate UK.)

Competing interests statement

GSB and DA are employed by Neurofenix (United Kingdom), a digital therapeutics company developing a therapy platform and sensor-based devices to augment rehabilitation. Neurofenix provided the Neurofenix platforms and technical support to the research therapists. Neurofenix had no influence on the design of the study, data collection, analysis and interpretation of the data or manuscript preparation. KB and DS were employed by Brunel University London during the period of the study, and are now Neurofenix employees.

All other authors declare no competing interests.

Patient consent

Obtained.

For peer review only - http://bmjopen.bmj.com/site/about/guidelines.xhtml

Ethics approval

Ethical approval was granted by the College of Health and Life Sciences Research Ethics Committee (REC) in Brunel University London (10249-MHR-Mar/2018-12322-2). Participants gave informed consent before taking part.

Provenance and peer review

Not commissioned; externally peer reviewed.

Data statement

Anonymised aggregated data will be made available in a public repository once the data have obtained validation through publication.

Participants did not consent for datasets to be stored or accessed outside of the research team. Therefore, no datasets have been made publicly available.

For peer review only

For peer review only - http://bmjopen.bmj.com/site/about/guidelines.xhtml

Reference List

1. Broeks JG, Lankhorst GJ, Rumping K, et al. The long-term outcome of arm function after stroke: results of a follow-up study. *Disabil Rehabil* 1999;21:357–64.

2. Nakayama H, Jørgensen HS, Raaschou HO, Olsen TS. Recovery of upper extremity function in stroke patients: the Copenhagen Stroke Study. *Arch Phys Med Rehabil* 1994;75(4):394-8.

3. Kwakkel G, Kollen BJ. Predicting activities after stroke: what is clinically relevant? *International J Stroke* 2013;8(1):25-32.

4. Lohse KR, Lang CE, Boyd LA. Is more better? Using Metadata to Explore Dose–Response Relationships in Stroke Rehabilitation. *Stroke* 2014;45:2053-2058.

5. Vratsistas-Curto A, Sherrington C, McCluskey A. Dosage and predictors of arm practice during inpatient stroke rehabilitation: an inception cohort study. *Disabil Rehabil* 2019;43(5):640-647.

6. Hayward KS, Brauer SG. Dose of arm activity training during acute and subacute rehabilitation post-stroke: A systematic review of the literature. *Clin Rehabil* 2015;29(12):1234-43.

7. Baniña MC, Molad R, Solomon JM, et al. Exercise intensity of the upper-limb can be enhanced using a virtual rehabilitation system. *Disabil Rehabil Assist Technol* 2020 DOI:10.1080/17483107.2020.1765421

8. Brunner I, Sture Skouen J, Hofstad H, et al. Is upper-limb virtual reality training more intensive than conventional training for patients in the subacute phase after stroke? An analysis of treatment intensity and content. *BMC Neurol* 2016;16:219. DOI 10.1186/s12883-016-0740-y.

9. Levac D, Glegg SM, Sveistrup H, et al. A knowledge translation intervention to enhance clinical application of a virtual reality system in stroke rehabilitation. *BMC Health Serv Res* 2016;16(1):557.

10. Wingham J, Adie K, Turner D, et al. Participant and caregiver experience of the Nintendo Wii SportsTM after stroke: qualitative study of the trial of WiiTM in stroke (TWIST). *Clin Rehabil* 2015;29(3):295-305.

11. Tsekleves E, Paraskevopoulos IT, Warland A, et al. Development and preliminary evaluation of a novel low cost VR-based upper-limb stroke rehabilitation platform using Wii technology. *Disabil Rehabil Assist Technol* 2016;11(5):413-22.

For peer review only - http://bmjopen.bmj.com/site/about/guidelines.xhtml

12. Eldridge SM, Chan CL, Campbell MJ, et al. CONSORT 2010 statement: extension to randomised pilot and feasibility trials. *BMJ* 2016; 355:i5239.

13. Kilbride C, Scott DJ, Butcher T, et al. Rehabilitation via HOMe Based gaming exercise for the Upper-limb post-stroke (RHOMBUS): protocol of an intervention feasibility trial. *BMJ Open*. 2018;8(11):e026620.

14. Bruno A, Akinwuntan AE, Lin C, et al. Simplified modified Rankin scale questionnaire: reproducibility over the telephone and validation with quality of life. *Stroke* 2011;42(8):2276-9.

15. Bruno A, Shah N, Akinwuntan AE, et al. Stroke size correlates with functional outcome on the simplified modified Rankin Scale questionnaire. *J Stroke Cerebrovasc Dis* 2013;22(6):781-3.

16. Sivan M, O'Connor RJ, Makower S, et al. Systematic review of outcome measures used in the evaluation of robot-assisted upper-limb exercise in stroke. *J Rehabil Med* 2011;43(3):181-9.

17. Van der Lee, JH, De Groot V, Beckerman H, et al. The intra-and interrater reliability of the action research arm test: a practical test of upper extremity function in patients with stroke. *Arch Phys Med Rehabil* 2001;82(1):14-9.

18. Yozbatiran N, Der-Yeghiaian L, Cramer SC. A standardized approach to performing the action research arm test. *Neurorehabil Neural Repair* 2008 Jan-Feb;22(1):78-90.

19. Page SJ, Fulk GD, Boyne P. Clinically important differences for the upper-extremity Fugl-Meyer Scale in people with minimal to moderate impairment due to chronic stroke. *Phys Ther* 2012;92(6):791-8.

20. Duncan PW, Propst M, Nelson SG. Reliability of the Fugl-Meyer assessment of sensorimotor recovery following cerebrovascular accident. *Phys Ther* 1983;63(10):1606-10.

21. Trigg R, Wood VA. The Subjective Index of Physical and Social Outcome (SIPSO): a new measure for use with stroke patients. *Clin Rehabil* 2000;14(3):288-99.

22. Simpson LA, Eng JJ. Functional recovery following stroke: capturing changes in upper-extremity function. *Neurorehabil Neural Repair* 2013;27(3):240-50.

23. Van der Lee, JH, Beckerman H, Knol DL, et al. Clinimetric properties of the motor activity log for the assessment of arm use in hemiparetic patients. *Stroke* 2004;35(6):1410-4.

For peer review only

For peer review only - http://bmjopen.bmj.com/site/about/guidelines.xhtml

24. Bohannon RW, Smith MB. Interrater reliability of a modified Ashworth scale of muscle spasticity. *Phys Ther* 1987;67(2):206-7.

25. van de Pol, RJ, van Trijffel E, Lucas C. Inter-rater reliability for measurement of passive physiological range of motion of upper extremity joints is better if instruments are used: a systematic review. *J Physiother* 2010;56(1):7-17.

26. Herdman M, Gudex C, Lloyd A, et al. Development and preliminary testing of the new five-level version of EQ-5D (EQ-5D-5L). *Qual Life Res* 2011;20(10):1727-36.

27. van Hout B, Janssen MF, Feng YS, et al. Interim scoring for the EQ-5D-5L: mapping the EQ-5D-5L to EQ-5D-3L value sets. *Value Health* 2012;15:708-15.

28. Rodgers H, Shaw L, Cant R, et al. Evaluating an extended rehabilitation service for stroke patients (EXTRAS): study protocol for a randomised controlled trial. *Trials* 2015;16(1):205.

29. Beecham J, Knapp M. Costing psychiatric interventions. *Measuring Mental Health Needs.* 2001;2:200-24.

30. Lerdal A, Kottorp A. Psychometric properties of the Fatigue Severity Scale—Rasch analyses of individual responses in a Norwegian stroke cohort. *Int J Nurs Stud* 2011;48(10):1258-65.

31. Demers L, Weiss-Lambrou R, Ska B. The Quebec user evaluation of satisfaction with assistive technology (QUEST 2.0): an overview and recent progress. *Technol Disabil* 2002;14:101-105.

32. Sim J, Lewis M. The size of a pilot study for a clinical trial should be calculated in relation to considerations of precision and efficiency. *J Clin Epidemiol* 2012;65:3:301-308.

33. SA Julious, NE McIntyre. Sample sizes for trials involving multiple correlated must-win comparisons. *Pharm Stat* 2012;11(2):177-185.

34. Gale NK, Heath G, Cameron E, et al. (2013) Using the framework method for the analysis of qualitative research data in multi-disciplinary health research. *BMC Med Res Methodol,* 2013;3,117 hyyp://www.biomedcentral.com/1471-2288/13/117.

35. Holloway I, Galvin K. Qualitative research in nursing and healthcare. John Wiley & Sons; 2016.

36. Laver KE, Lange B, George S, et al. Virtual reality for stroke rehabilitation. *Cochrane Database Syst Rev* 2017, Issue 11. Art. No.: CD008349. DOI: 10.1002/14651858.CD008349.pub4.

For peer review only

37. Adie K, Schofield C, Berrow M, et al. Does the use of Nintendo Wii SportsTM improve arm function? Trial of WiiTM in Stroke: a randomized controlled trial and economics analysis. *Clin Rehabil* 2017;31(2) 173–185. DOI: 10.1177/0269215516637893

38. Chen Y, Abel KT, Janecek JT, et al. Home-based technologies for stroke rehabilitation: A systematic review. *Int J Med Inform* 2019;123:11-22.

39. Schultz KF, Grimes DA Sample size slippages in randomised controlled trials: exclusions, the lost and the wayward. *Lancet* 2002;359:781-85.

40. Standen PJ, Threapleton K, Connell L, et al. Patients' use of a home-based virtual reality system to provide rehabilitation of the upper-limb following stroke. *Phys Ther* 2015;95:350-359.

41. Kwakkel G, Lannin NA, Borschmann K, et al. Standardized measurement of sensorimotor recovery in stroke trials: consensus-based core recommendations from the Stroke Recovery and Rehabilitation Roundtable. *Neurorehabil Neural Repair* 2017;31(9):784–792.

42. Syed UE, Kamal A. Video game-based and conventional therapies in patients of neurological deficits: an experimental study. *Disabil Rehabil Assist Technol* 2019;DOI: 10.1080/17483107.2019.1679266.

43. Lehmann I, Baer G, Schuster-Amft C. Experience of an upper-limb training program with a non-immersive virtual reality system in patients after stroke: a qualitative study. *Physiother* 2020;107:317-326.

44. Barrett N, Swain I, Gatzidis C, et al. The use and effect of video game design theory in the creation of game-based systems for upper-limb stroke rehabilitation. *J Rehabil Assist Technol Eng* 2016;3:1-16.

45. National Institute for Health and Care Excellence Guideline. Stroke and transient ischaemic attack in over 16s: diagnosis and initial management. NICE Guideline [NG128]:2019 https://www.nice.org.uk/guidance/ng128.

46. Domínguez-Téllez P, Moral-Muñoz JA, Salazar A, et al. Game-based virtual reality interventions to improve upper-limb motor function and quality of life after stroke: systematic review and meta-analysis. *Games Health J: Research, Development, and Clinical Applications.* 2020;9:1.

For peer review only - http://bmjopen.bmj.com/site/about/guidelines.xhtml

47. Pezzera M, Borghese NA. Dynamic difficulty adjustment in exer-games for rehabilitation: a mixed approach. IEEE 8th International Conference on Serious Games and Applications for Health (SeGAH), 2020:DOI 10.1109/SeGAH49190.2020.9201871.

For peer review only

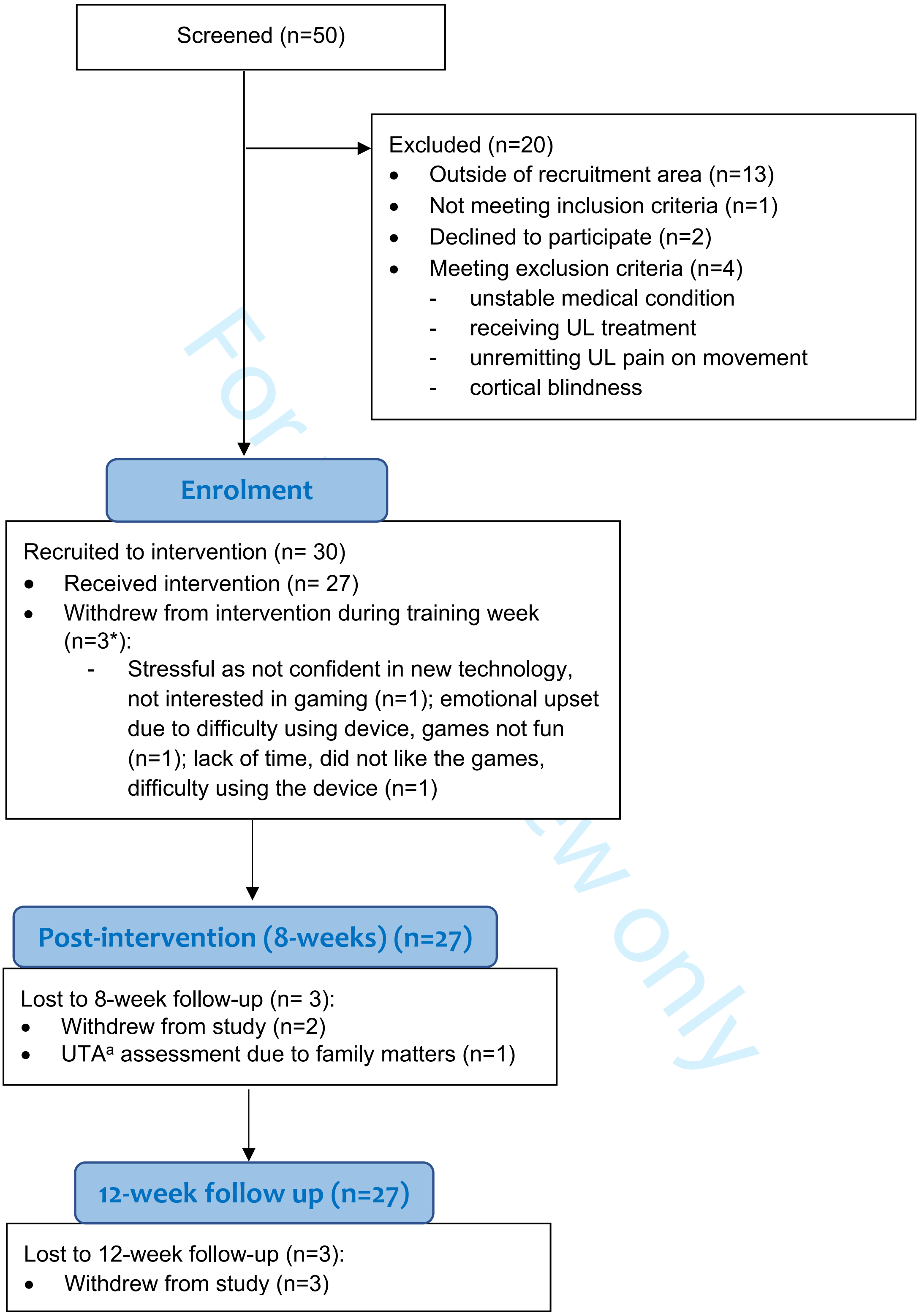

Figure 1. CONSORT flow of participants through the trial.
[a]Unable to attend.

CONSORT 2010 checklist of information to include when reporting a pilot or feasibility trial*

Section/Topic	Item No	Checklist item	Reported on page No
**Title and abstract**			
	1a	Identification as a pilot or feasibility randomised trial in the title	1
	1b	Structured summary of pilot trial design, methods, results, and conclusions (for specific guidance see CONSORT abstract extension for pilot trials)	2,3
**Introduction**			
Background and objectives	2a	Scientific background and explanation of rationale for future definitive trial, and reasons for randomised pilot trial	5
	2b	Specific objectives or research questions for pilot trial	5
**Methods**			
Trial design	3a	Description of pilot trial design (such as parallel, factorial) including allocation ratio	6
	3b	Important changes to methods after pilot trial commencement (such as eligibility criteria), with reasons	n/a
Participants	4a	Eligibility criteria for participants	6
	4b	Settings and locations where the data were collected	6
	4c	How participants were identified and consented	6
Interventions	5	The interventions for each group with sufficient details to allow replication, including how and when they were actually administered	6,7
Outcomes	6a	Completely defined prespecified assessments or measurements to address each pilot trial objective specified in 2b, including how and when they were assessed	7,8
	6b	Any changes to pilot trial assessments or measurements after the pilot trial commenced, with reasons	n/a
	6c	If applicable, prespecified criteria used to judge whether, or how, to proceed with future definitive trial	n/a
Sample size	7a	Rationale for numbers in the pilot trial	9
	7b	When applicable, explanation of any interim analyses and stopping guidelines	n/a
Randomisation:			
Sequence generation	8a	Method used to generate the random allocation sequence	n/a
	8b	Type of randomisation(s); details of any restriction (such as blocking and block size)	n/a
Allocation concealment mechanism	9	Mechanism used to implement the random allocation sequence (such as sequentially numbered containers), describing any steps taken to conceal the sequence until interventions were assigned	n/a

For peer review only

Implementation	10	Who generated the random allocation sequence, who enrolled participants, and who assigned participants to interventions	n/a
Blinding	11a	If done, who was blinded after assignment to interventions (for example, participants, care providers, those assessing outcomes) and how	n/a
	11b	If relevant, description of the similarity of interventions	n/a
Statistical methods	12	Methods used to address each pilot trial objective whether qualitative or quantitative	9,10
**Results**			
Participant flow (a diagram is strongly recommended)	13a	For each group, the numbers of participants who were approached and/or assessed for eligibility, randomly assigned, received intended treatment, and were assessed for each objective	11, Fig 1
	13b	For each group, losses and exclusions after randomisation, together with reasons	11, Fig 1
Recruitment	14a	Dates defining the periods of recruitment and follow-up	10
	14b	Why the pilot trial ended or was stopped	n/a
Baseline data	15	A table showing baseline demographic and clinical characteristics for each group	11
Numbers analysed	16	For each objective, number of participants (denominator) included in each analysis. If relevant, these numbers should be by randomised group	12, 13
Outcomes and estimation	17	For each objective, results including expressions of uncertainty (such as 95% confidence interval) for any estimates. If relevant, these results should be by randomised group	19
Ancillary analyses	18	Results of any other analyses performed that could be used to inform the future definitive trial	13
Harms	19	All important harms or unintended effects in each group (for specific guidance see CONSORT for harms)	14,17
	19a	If relevant, other important unintended consequences	n/a
**Discussion**			
Limitations	20	Pilot trial limitations, addressing sources of potential bias and remaining uncertainty about feasibility	4, 20-22,
Generalisability	21	Generalisability (applicability) of pilot trial methods and findings to future definitive trial and other studies	22
Interpretation	22	Interpretation consistent with pilot trial objectives and findings, balancing potential benefits and harms, and considering other relevant evidence	20-22,
	22a	Implications for progression from pilot to future definitive trial, including any proposed amendments	22
**Other information**			
Registration	23	Registration number for pilot trial and name of trial registry	3
Protocol	24	Where the pilot trial protocol can be accessed, if available	5
Funding	25	Sources of funding and other support (such as supply of drugs), role of funders	23
	26	Ethical approval or approval by research review committee, confirmed with reference number	24

For peer review only

Citation: Eldridge SM, Chan CL, Campbell MJ, Bond CM, Hopewell S, Thabane L, et al. CONSORT 2010 statement: extension to randomised pilot and feasibility trials. BMJ. 2016;355.

*We strongly recommend reading this statement in conjunction with the CONSORT 2010, extension to randomised pilot and feasibility trials, Explanation and Elaboration for important clarifications on all the items. If relevant, we also recommend reading CONSORT extensions for cluster randomised trials, non-inferiority and equivalence trials, non-pharmacological treatments, herbal interventions, and pragmatic trials. Additional extensions are forthcoming: for those and for up to date references relevant to this checklist, see www.consort-statement.org.

For peer review only

Safety, feasibility, acceptability, and preliminary effects of the Neurofenix Platform for Rehabilitation via HOMe Based gaming exercise for the Upper-limb post-stroke (RHOMBUS): results of a feasibility intervention study.

Journal:	*BMJ Open*
Manuscript ID	bmjopen-2021-052555.R1
Article Type:	Original research
Date Submitted by the Author:	13-Jan-2022
Complete List of Authors:	Kilbride, Cherry; Brunel University London, Department of Health Sciences<br>Scott, Daniel J M; Brunel University London, Department of Health Sciences; Neurofenix<br>Butcher, Tom; Brunel University College of Health and Life Sciences, Health Sciences<br>Norris, Meriel; Brunel University London, College of Health and Life Sciences<br>Warland, Alyson; Brunel University Department of Clinical Sciences, Clinical Sciences<br>Anokye, Nana; Brunel University London, Department of Health Sciences<br>Cassidy, Elizabeth ; Freelance academic and research supervisor<br>Baker, Karen; Brunel University London, Department of Health Sciences; Neurofenix<br>Athanasiou, Dimitrios; Neurofenix<br>Singla-Buxarrais, Guillem; Neurofenix<br>Nowicky, Alexander; Brunel University London, Department of Health Sciences<br>Ryan , Jennifer ; Royal College of Surgeons in Ireland, Public Health and Epidemiology
**Primary Subject Heading**:	Rehabilitation medicine
Secondary Subject Heading:	Rehabilitation medicine, Qualitative research, Evidence based practice
Keywords:	Stroke < NEUROLOGY, REHABILITATION MEDICINE, Adult neurology < NEUROLOGY

I, the Submitting Author has the right to grant and does grant on behalf of all authors of the Work (as defined in the below author licence), an exclusive licence and/or a non-exclusive licence for contributions from authors who are: i) UK Crown employees; ii) where BMJ has agreed a CC-BY licence shall apply, and/or iii) in accordance with the terms applicable for US Federal Government officers or employees acting as part of their official duties; on a worldwide, perpetual, irrevocable, royalty-free basis to BMJ Publishing Group Ltd ("BMJ") its licensees and where the relevant Journal is co-owned by BMJ to the co-owners of the Journal, to publish the Work in this journal and any other BMJ products and to exploit all rights, as set out in our licence.

The Submitting Author accepts and understands that any supply made under these terms is made by BMJ to the Submitting Author unless you are acting as an employee on behalf of your employer or a postgraduate student of an affiliated institution which is paying any applicable article publishing charge ("APC") for Open Access articles. Where the Submitting Author wishes to make the Work available on an Open Access basis (and intends to pay the relevant APC), the terms of reuse of such Open Access shall be governed by a Creative Commons licence – details of these licences and which Creative Commons licence will apply to this Work are set out in our licence referred to above.

Other than as permitted in any relevant BMJ Author's Self Archiving Policies, I confirm this Work has not been accepted for publication elsewhere, is not being considered for publication elsewhere and does not duplicate material already published. I confirm all authors consent to publication of this Work and authorise the granting of this licence.

For peer review only

Safety, feasibility, acceptability, and preliminary effects of the Neurofenix Platform for Rehabilitation via HOMe Based gaming exercise for the Upper-limb post-stroke (RHOMBUS): results of a feasibility intervention study.

Authors
Cherry Kilbride
Department of Health Sciences, Institute of Health, Medicine and Environment, Brunel University London, Uxbridge, UK
Daniel J M Scott
Department of Health Sciences, Institute of Health, Medicine and Environment, Brunel University London, Uxbridge, UK; Neurofenix, London, UK
Tom Butcher
Department of Health Sciences, Institute of Health, Medicine and Environment, Brunel University London, Uxbridge, UK
Meriel Norris
Department of Health Sciences, Institute of Health, Medicine and Environment, Brunel University London, Uxbridge, UK
Alyson Warland
Department of Health Sciences, Institute of Health, Medicine and Environment, Brunel University London, Uxbridge, UK
Nana Anokye
Department of Health Sciences, Institute of Health, Medicine and Environment, Brunel University London, Uxbridge, UK
Elizabeth Cassidy
Freelance Academic, London
Karen Baker
Department of Health Sciences, Institute of Health, Medicine and Environment, Brunel University London, Uxbridge, UK; Neurofenix, London, UK
Dimitrios A Athanasiou
Neurofenix, London, UK
Guillem Singla-Buxarrais
Neurofenix, London, UK
Alexander Nowicky
Department of Health Sciences, Institute of Health, Medicine and Environment, Brunel University London, Uxbridge, UK
Jennifer Ryan
Department of Public Health and Epidemiology, RCSI University of Medicine and Health Sciences, Dublin, Ireland; Department of Health Sciences, Institute of Health, Medicine and Environment, Brunel University London, Uxbridge, UK

Correspondence to:
Cherry Kilbride: cherry.kilbride@brunel.ac.uk
+44 (0)1895 268675
Brunel University London, Department of Health Sciences, Uxbridge UB8 3PH

Version 2

DATE: 11 December 2021

WORD COUNT (excluding title page, abstract, references, figures and tables): 4087

For peer review only - http://bmjopen.bmj.com/site/about/guidelines.xhtml

Abstract

Objectives

To investigate the safety, feasibility and acceptability of the Neurofenix platform for home-based rehabilitation of the upper-limb (UL).

Design

A non-randomised intervention design with a parallel process evaluation.

Setting

Participants' homes South-East England.

Participants

Thirty adults (≥18 years), minimum 12-weeks post-stroke, not receiving UL rehabilitation, scoring 9-25 on the Motricity Index (elbow and shoulder), with sufficient cognitive and communicative abilities to participate.

Interventions

Participants were trained to use the platform, followed by one-week of graded game-play exposure and six-weeks training, aiming for a minimum 45 minutes, 5 days/week.

Outcomes

Safety was determined by assessing pain and post-stroke fatigue at 8 and 12-weeks, and adverse events (AEs). Impairment, activity and participation outcomes were measured. Intervention feasibility was determined by amount of specialist training and support required to complete the intervention, time and days spent training, and number of UL movements performed. Acceptability was assessed by a satisfaction questionnaire and semi-structured interviews.

Results

Participants [14 women; mean (SD) age 60.0 (11.3) years] were a median of 4.9 years post-stroke (minimum-maximum: 1-28 years). Twenty-seven participants completed the intervention. The odds of having shoulder pain were lower at 8-weeks (OR 0.45, 95% CI 0.24 to 0.83, p=0.010) and 12-weeks (OR 0.46, 95% CI 0.25 to 0.86, p=0.014) compared to baseline. Fugl-Meyer upper extremity, Motor Activity Log and passive range of movement improved. No other gains were recorded. Post-stroke fatigue did not change. Thirty mild and short-term AEs and 1 serious (unrelated) AE were reported by 19 participants. Participants trained with the platform for a median of 17.4 hours over 7-weeks (minimum-maximum: 0.3-46.9 hours), equating to a median of 149 minutes per week. The median satisfaction score was 36 out of 40.

For peer review only - http://bmjopen.bmj.com/site/about/guidelines.xhtml

For peer review only

Conclusion

The Neurofenix platform is a safe, feasible and well accepted way to support UL training for people at least three months post-stroke.

Ethics approval

Ethics approval granted from Brunel University London (10249-MHR-Mar/2018-12322-2).

Trial registration number

ISRCTN60291412 (doi 10.1186/ISRCTN60291412). Protocol version:1.0; pre-results.

Funding

Innovate UK.

For the BMJ Open Submission website (ScholarOne)

Keywords

Rehabilitation, stroke, upper-limb, virtual reality, gaming, protocol, feasibility.

Keywords (MeSH)

Stroke therapy/rehabilitation/methods

Upper extremity/limb

Virtual reality stroke rehabilitation

Video games

Feasibility

For peer review only - http://bmjopen.bmj.com/site/about/guidelines.xhtml

Article Summary

Strengths and limitations of this study

1. A feasibility trial developed following Medical Research Council Framework for the development of complex interventions.
2. Mixed-method approach for answering feasibility and acceptability objectives.
3. Fidelity activity data were objectively measured through the platform.
4. Inclusive of people with higher levels of UL impairment.
5. No control group or blinded assessor.

For peer review only

For peer review only - http://bmjopen.bmj.com/site/about/guidelines.xhtml

Introduction

Upper-limb (UL) dysfunction is a common and major contributor to physical disability following stroke.[1] Approximately two-thirds of stroke survivors experience upper limb impairment [2] and only 5% to 20% achieve full UL recovery at six months post-stroke.[3]

High intensity UL stroke rehabilitation improves upper limb recovery with a positive dose-response relationship for individuals in the acute and chronic phases after stroke.[4] UL training provided in rehabilitation settings typically falls below the hundreds of repetitions considered necessary to drive functional recovery.[5,6]

Virtual reality (VR) platforms using interactive computer-based games have emerged as a feasible and motivating way of supporting UL rehabilitation at intensities significantly higher than conventional therapy.[7,8] However, bespoke VR devices are often unwieldly, complex, and expensive and while commercially available devices may be more accessible, they often demand sophisticated movement combinations which are not possible for many stroke survivors to perform.[9-11]

The Neurofenix platform is a novel, affordable and portable VR device specifically developed by stroke survivors, specialist neuro-physiotherapists and bioengineers for gamification of post-stroke UL rehabilitation (www.neurofenix.com). The platform enables and encourages stroke survivors to exercise their arm and hand at home with little or no assistance. The overall aim of this study was to investigate the safety, feasibility and acceptability of the platform for home-based rehabilitation of the UL. Additional objectives were (1) to assess the feasibility of conducting a definitive trial of the clinical and cost-effectiveness of the intervention, (2) to understand factors relating to people with stroke and the intervention that may impact on fidelity to the intervention, and (3) to examine preliminary effects of the intervention.

Methods

The study is reported in accordance with guidance on reporting pilot and feasibility studies.[12] Further details on trial procedures are detailed in the protocol.[13]

Patient and Public Involvement

Stroke survivors were involved in the iterative development of the Neurofenix platform. Two further stroke survivors acted in an advisory capacity during the study, providing input to the protocol and trial documentation, and dissemination.

For peer review only - http://bmjopen.bmj.com/site/about/guidelines.xhtml

Modifications to study design since trial registration

1. At the time of trial registration, the intervention was called Gameball device; it has since been renamed the Neurofenix Platform.
2. Measurement of pain – simplified to self-report using VAS 0-10 with a focus on shoulder pain.
3. Removal of EQ5D-L overall health on the day VAS 0-100
4. Addition of data analysis for preliminary effects of intervention (https://www.nihr.ac.uk/documents/nihr-research-for-patient-benefit-rfpb-programme-guidance-on-applying-for-feasibility-studies/20474)

For peer review only

Trial Design

The RHOMBUS study used a non-randomised intervention design with a parallel process evaluation. Participants were familiarised, set-up and trained to use the device by a research therapist, followed by one-week of graded exposure to game-play, and subsequent six-week independent training phase. The device was removed at the end of the 7-week training period. Assessments were performed at baseline, 8, and 12-weeks.

Participants

Participants were recruited from a university stroke database, via gatekeepers for three community stroke groups and the ISRCTN Registry website between April and September 2018. Inclusion criteria were: 1) aged 18 years or over; 2) capacity to consent; 3) self-reported diagnosis of stroke; 4) 12-weeks minimum post-stroke and not in receipt of UL rehabilitation; 5) mild-to-severe reduction in arm function post-stroke (Motricity Index (MI) score between 9-25 for elbow and shoulder movement); 6) able to sit or stand independently, with or without an aid, for a minimum of 5 minutes; and 7) sufficient English to participate in the intervention and assessments. Exclusion criteria were: 1) unstable medical conditions; 2) uncontrolled photosensitive epilepsy; 3) acquired brain injury from other causes, bilateral or cerebellar lesions; 4) uncompensated visual neglect, hemianopia or uncorrected visual field deficits; and 5) pre-existing, unremitting arm pain at rest. All participants provided written informed consent.

Intervention

The intervention is described in detail in the protocol.[13] Briefly, the Neurofenix platform is a digital device consisting of non-immersive VR software in the form of an App on a tablet and Bluetooth-connected hand controller (NeuroBall) and arm controllers (Neurobands). The NeuroBall is secured

For peer review only - http://bmjopen.bmj.com/site/about/guidelines.xhtml

to the impaired hand through a system of straps and elastic finger holds. Motion sensors within the NeuroBall detect UL movements and translate these to control games displayed on a tablet. For those without sufficient strength and/or range of movement in the hand and wrist to control the NeuroBall, the NeuroBands are used as an alternative. The NeuroBands are two small motion sensors; the first straps around the upper arm and the second around either the forearm or the hand. If the second NeuroBand is placed on the forearm, then elbow flexion and extension can be used to control the games, and if placed around the hand, wrist flexion and extension can be used. Participants can choose from seven games specifically developed for UL rehabilitation (see www.neurofenix.com).

Two research therapists attended two half-day training sessions on technical and operational procedures relating to the device. Neurofenix engineers provided additional technical support to the therapists and participants throughout the study if required.

Following the baseline assessment, a research therapist attended the participant's home. The visit commenced with a brief questionnaire about the participant's prior experience with technology and video games and confidence in using new technology. The pace and depth of subsequent teaching was based on the participant's experience and confidence. The participant was then given the Neurofenix platform consisting of a NeuroBall or two NeuroBands, a tablet, a tablet stand, chargers, a handbook, and a one-page "Quickstart" guide.

A research therapist trained the participant in how to use the platform independently or with the help of a carer if requested. Specifically, participants were taught safety precautions and were trained to (1) turn the device and tablet on and off, (2) don and doff the device, (3) calibrate the device, (4) play each of the games on the tablet, (5) navigate the menus, (6) track their progress, (7) use the handbook and (8) charge the devices. The research therapist advised each participant on the starting duration and frequency of use based on signs of pain and post-stroke fatigue during the visit. As participants were not receiving UL rehabilitation at the time of the study, muscle ache as a normal response to training was discussed with the stroke survivor. Participants were advised to gradually increase the amount of use over the first week, with the aim of achieving a minimum of 45 minutes of training on 5 days a week, and to continue with this duration and frequency for the subsequent 6-weeks, increasing the dose if able. Participants independently chose which games to play during the intervention. Participants received two scheduled phone calls from the research therapists during week -1 and week-3 to ensure no clinical problems or technical faults had occurred. After this second call, participants had no input from the research team, but were advised to contact the team if they needed clinical or technical support. Participants were contacted at the

For peer review only - http://bmjopen.bmj.com/site/about/guidelines.xhtml

end of 7-weeks, asked to stop using the device, and arranged a time for the therapist to collect the device.

Procedures

Two research therapists completed assessments in participants' homes. Socioeconomic characteristics, stroke-specific characteristics and global disability were measured using the simplified modified Rankin Scale questionnaire (smQRs); higher score represents greater disability (range 1-5).[14,15]

Outcomes

Arm and hand function were assessed objectively using the Action Research Arm Test (ARAT), higher scores indicate better function.[16-18] UL impairment was assessed using the Fugl-Meyer Assessment–upper-limb (FMA–UE); higher scores indicate less impairment.[19,20] Participation was measured on the 10-item Subjective Index of Physical and Social Outcome (SIPSO); higher scores signify increased ability to reintegrate to a 'normal' lifestyle.[21] Self-reported arm use during functional tasks was measured using the 28-item Motor Activity Log (MAL); higher scores indicate more activity.[22,23]

The Modified Modified Ashworth Scale (MMAS) was used to assess spasticity of the shoulder adductors and internal rotators, elbow, wrist and finger flexors.[24] The highest spasticity score within the UL was selected for analysis. Passive range of movement (PROM) of the shoulder (flexion, abduction, external rotation), elbow (flexion and extension) and wrist (extension) were assessed using goniometry.[25] Quality of life was assessed using the EQ-5D-5L questionnaire.[26] A utility value [i.e. a score] was calculated as recommended using the cross-walk function which maps the EQ-5D-3L and newer EQ-5D-5L questionnaires] ;[27] a higher value indicates better quality of life. A modified version of the Client Service Receipt Inventory (CSRI) was used to assess health service use.[28,29]

Safety

Safety was determined by assessing pain, post-stroke fatigue and adverse events (AEs). At 8- and 12-weeks, participants were asked if they experienced shoulder pain over the past 7 days, and if so to rate the intensity of shoulder pain on a 10-point visual analogue scale (VAS), with higher scores indicating more pain. Post-stroke fatigue level was assessed using the Fatigue Severity Scale (FSS-7).[30] At the 8- and 12-week assessment, participants were asked if they had experienced an AE since their last contact with the research team using standardised questioning. An AE is any untoward medical occurrence affecting a participant during the study which may or may not be

For peer review only - http://bmjopen.bmj.com/site/about/guidelines.xhtml

related to the study intervention i.e. an AE is unrelated if there is no evidence of a causal relationship with the trial intervention and another documented cause of the AE is most plausible. A serious AEs (SAEs) is an AE that results in death, is life threatening, requires hospitalisation or results in a disability or incapacity.

Feasibility

Feasibility of the intervention was determined by recording the number of people who received the training sessions, the number and duration of sessions required, and the number and duration of clinical or technical phone calls and home visits required. Post-training confidence with the Neurofenix Platform was assessed with a 10-point VAS (higher score equates to more confidence).

Feasibility was also determined by assessing fidelity to the intervention. Fidelity was assessed as time spent training the UL with the platform, number of days training, and number of UL movements performed. Sensors were used to automatically collect data from the Neurofenix platform, which was stored on the participant's tablet until remotely downloaded by a member of the research team.

Acceptability

The Quebec User Evaluation of Satisfaction with Assistive Technology (QUEST 2.0) [31] was completed at 8-weeks to assess user satisfaction of the device; higher scores indicate higher satisfaction. Semi-structured interviews were completed with a purposive sample (male, female, high and low user engagement) of 18 participants to explore the acceptability of the intervention, and the experience of taking part in the study. Interviews were conducted by a research therapist (typically who did not provide training to that participant) and the analysis was undertaken by a member of the research team who did not otherwise have contact with participants (EC). Carers or spouses involved in the intervention were also invited to take part as a dyad.

Feasibility of a definitive trial

The feasibility of conducting a definitive trial was assessed by examining recruitment and retention rates, outcome measure completion and reasons for missing data associated with the data collection tools.

Sample Size

We included 30 participants based on recommendations that between 24 and 50 participants are sufficient for feasibility studies.[32,33]

Data analysis

For peer review only - http://bmjopen.bmj.com/site/about/guidelines.xhtml

Table 1. Baseline characteristics of RHOMBUS participants

	n (%)	Mean (SD)	Median (IQR),	Min-max
Age, year	30	60 (11.3)	60 (12),	36 to 85
Women	14 (47)	-	-	

Distribution of data was explored using Q-Q plots, histograms and cross-tabulations. Descriptive statistics (e.g., mean, standard deviation, median, interquartile range) were used to report data. Generalised estimating equations with an exchangeable working matrix and robust standard errors were used to examine change between baseline and follow-up assessments on the VAS, FSS, ARAT, FMA-UE, MMAS, MAL-28 (amount of use score only), SIPSO and goniometry. A generalised estimating equation with a logit link, exchangeable working matrix and robust standard errors was used to examine if the odds of shoulder pain changed over time.

Associations between total time spent exercising the UL with the platform, number of repetitions performed and age, ARAT score at baseline, FMA-UE score at baseline, confidence with new technology at baseline, post-training confidence with the Neurofenix platform were examined using Spearman's correlations. Associations between total time spent exercising the UL with the platform, number of repetitions performed, and gender were examined using Mann Whitney U tests.

Qualitative data from the semi-structured interviews were analysed using the five stage Framework Analysis method.[34] Framework Analysis incorporates both deductive and inductive coding and enabled trial processes and experiences to be explored and reported. This method provides a strong audit trail of the analytical process, which enhances transparency.[35] One person (EC) independent to the delivery team led the analysis. Four researchers (EC, MN, TB, DS) independently coded the same three transcripts, then discussed and agreed codes. A coding framework was then developed and used to code all the interviews.

Results

Thirty participants were recruited to the study between April and July 2018 (Figure 1). Participants (14 women) were mean (SD) 60.0 (11.3) years (minimum-maximum: 36-85 years), and a median of 4.9 years post-stroke (minimum-maximum: 1-28 years) (Table 1). The majority scored 2 or 3 on the smRSq. Mean (SD) FMA-UE score was 33 (16.4) and median (IQR) ARAT score was 8 (20).

Insert Figure 1 near here.

For peer review only - http://bmjopen.bmj.com/site/about/guidelines.xhtml

**Ethnicity**				
White	15 (50)	-	-	
Asian	12 (40)	-	-	
Black	2 (7)	-	-	
Mixed	1 (3)			
Stroke chronicity, year	30	5.5 (5.4)	4.9 (4.5)	1 to 28
Stroke type (self-report)				
Haemorrhagic	17 (57)			
Ischaemic	12 (40)			
Not known	1 (3)			
**smRSq**				
1.0	3 (10)			
2.0	11 (36)			
3.0	13 (43)			
4.0	1 (3)			
5.0	2 (6)			
FMA-UE (0-66)	30	33 (16.4)	33 (31),	8 to 63
ARAT (0-57)	30	16 (17.5)	8 (20),	0 to 57
MMAS worse score (0-5)	30	2 (0.98)	2 (2),	0 to 3
NIHSS	30	4.1 (3.1)	3 (3),	1 to 14
Played computer games before stroke (Yes)	19 (63)	-	-	
Played computer games since stroke (Yes)	15 (50)	-	-	
Owned a tablet, computer, console or smartphone (Yes)	28 (93)	-	-	
Confidence pre training with new technology (0-10)	30	5.63 (2.7)	5 (4)	
Confidence with NeuroPlatform post training (0-10)	29*	7.6 (2.24)	8 (3)	

*One participant withdrew during training. SD: standard deviation; IQR: inter quartile range; smRSq: simplified modified Rankin scale questionnaire; FMA-UE: Fugl Meyer upper extremity; ARAT: action research arm test; MMAS: modified modified Ashworth scale; NIHSS: national institute of health stroke scale.

For peer review only

Safety

The odds of having shoulder pain were lower at 8-weeks (OR 0.45, 95% CI 0.24 to 0.83, p=0.010) and 12-weeks (OR 0.46, 95% CI 0.25 to 0.86, p=0.014) compared to baseline. Mean intensity of shoulder pain, as assessed on a VAS, was lower at 12-weeks compared to baseline (mean difference -0.69, 95% CI -1.37 to 0.004, p=0.052; Table 2). There was no change in the level of post-stroke fatigue as measured by the FSS-7 score, at 8 or 12-weeks in comparison to baseline (Table 2). Thirty AEs and 1 SAE were reported by a total of 19 participants (Table 3). Nineteen AEs were deemed probably or possibly related to the intervention; the AEs were mild and short-term and no participants withdrew due to an AE. Eleven unrelated AEs (including four falls) were deemed to have no causal relationship with the trial procedures as determined by the Principle Investigator (CK) and in line with the principles of Good Clinical Practice (https://www.hra.nhs.uk/planning-and-improving-research/policies-standards-legislation/good-clinical-practice/).

Feasibility

Thirty participants completed the training session (Table 4). Twenty-five participants needed one training session, three needed two sessions, and two needed a third session. The median time for training, over 1-3 sessions, was 98 minutes per participant (minimum-maximum 70-290 minutes). Median (IQR) participant confidence with the device after the initial training session was 8 (2) out of 10. Nine participants (30%) contacted the research team for technical support during the intervention period. Five participants (17%) contacted the research team for clinical support. The total number of visits, calls and their median durations are reported in Table 5.

Fidelity to the intervention

Participants trained with the Neurofenix platform for a median (minimum-maximum) of 17.4 (0.3-46.9) hours over 7-weeks, equating to a median of 149 minutes per week. Participants trained for a mean (SD) of 25.4 (15.9) days over 7-weeks (minimum-maximum: 1-48 days), equating to mean (SD) 3.6 (2.3) days per week. Eight participants (26.7%) achieved at least 225 minutes per week during the study, which equates to the recommended 45 minutes of training per day on five days per week. Participants performed a median (minimum-maximum) of 15,092 (222-43,999) UL movements over 7-weeks.

No association was found between time spent training over 7-weeks and age (rho=0.24, p=0.203), ARAT (rho=0.06, p=0.744), FMA-UE scores (rho=0.003, p=0.987), confidence with new technology (rho=0.13, p = 0.505), confidence post-training (rho=-0.05, p=0.784). There was also no difference in time spent training between men and women (p=0.061). No association was found between number

For peer review only - http://bmjopen.bmj.com/site/about/guidelines.xhtml

of UL movements over 7-weeks and age (rho=0.18, p=0.351), ARAT (rho=0.10, p=0.614), FMA-UL (rho=0.06, p=0.761), confidence with new technology (rho=0.15, p=0.427), confidence post-training (rho=-0.005, p=0.979). There was also no difference in number of UL movements between men and women (p=0.105).

Acceptability

The median score on the Quebec User Evaluation of Satisfaction with assistive Technology (QUEST) was 36 out of 40 (range 21 to 40). The device was well received and provided motivating UL training (Box 1). Motivating factors included enjoyable games, positive in-game feedback, competition, and challenge, engagement with more purposeful games than prescribed home exercises. Participants suggested that a greater variety of games and more challenging levels might enhance engagement with the platform. The home-based self-directed training provided by the device and its compact size was positively appraised because it offered a structured practice schedule and set amount of time to train and enabled practice to be planned around daily life.

Preliminary effects

The mean difference in outcomes between baseline and 8 and 12-weeks respectively, is reported in Table 6. Scores on the FMA-UE (mean difference 2.68, 95% CI 0.86 to 4.49; p=0.004) and MAL (mean difference 0.19, 95% CI 0.06 to 0.32; p=0.004) improved between baseline and 8-weeks. SIPSO total score increased between baseline and 12-weeks (mean difference 2.17, 95% CI 0.32 to 4.01; p=0.021). Passive range of shoulder external rotation, elbow flexion and wrist extension increased between baseline and 8-weeks, and between baseline and 12-weeks ($p<0.05$; Table 6). No changes were found for other outcomes.

Feasibility of conducting a definitive trial

Fifty people expressed interest in participating in the study. Twenty were excluded for reasons outlined in Figure 1, resulting in 60% being recruited. Three people withdrew from the intervention; reasons for withdrawing are shown in Figure 1. All participants completed all outcome measures at baseline. Of the three people who withdrew from the intervention, two withdrew from the study and did not complete assessments at 8 or 12 weeks. One additional person did not complete the 8-week and 12-week assessment, respectively, resulting in 90% retention at follow-up assessments. In addition to the 10% missing data due to loss to follow-up, the FSS-7 was not assessed at 8-weeks, and shoulder external rotation was not assessed for one person at 12-weeks due to human error, and one person refused to complete the ARAT at 12-weeks. Data about medication dosage in the modified CSRI was missing for four participants.

For peer review only - http://bmjopen.bmj.com/site/about/guidelines.xhtml

Table 2. Change in scores on 7 Item Fatigue Severity Scale (FFS-7) and Visual Analogue Scale for pain (VAS) between baseline and 8, and baseline and 12-weeks.

	Baseline (n=30)	8-weeks (n=27)	Change; 8-weeks – baseline (n=27)	12-weeks (n=27)	Change; 12-weeks – baseline (n=27)
	Mean (SD)	Mean (SD)	Mean difference (95% CI); p value	Mean (SD)	Mean difference (95% CI); p value
Fatigue FSS-7[a] (7-49)	28.5 (13.2)	25.8 (13.9)	-1.59 (-6.51 to 3.34); 0.528	26.1 (12.5)	-1.98 (-5.75 to 1.79); 0.304
VAS-pain (0-10)	2 (2.24)	1.7 (2.10)	-0.10 (-.73 to 0.53); 0.756	1.22 (1.60)	-0.69 (-1.37 to 0.004); 0.052

[a]n=26 for 8-weeks

For peer review only - http://bmjopen.bmj.com/site/about/guidelines.xhtml

Table 3. Adverse events and serious adverse events reported during study

Event Type	Number of events	Attribution			People reporting event, (n)
		Number of probably related events	Number of possibly related events	Number of unrelated events	
SAE	1	0	0	1	1
AE	30	17[a]	2[b]	11[c]	19

[a]muscle fatigue/soreness/discomfort (n=9); eye strain (n=2); upper-limb pain (n=4), self-reported increase in spasticity (n=2)
[b]neck pain (n=2)
[c]fatigue (n=2); falls (n=4); blurred vision (n=1); headache (n=4)

For peer review only

Table 4. Training sessions required per participants and duration of training

	Number of participants receiving, n (%)	Duration, minutes		
		Mean (SD)	Median (IQR)	Minimum-maximum
**Training sessions**				
First training session	30 (100)	95.23 (19.5)	92 (25)	55-150
Second training session	3 (10)	48.33 (35.5)	55 (70)	10-80
Third training session	2 (6.7)	95.0 (7.1)	95 (10)	90-100

IQR: interquartile range; SD: standard deviation

Table 5. Total number of phone calls and home visits (technical and clinical)

	Number of calls/visits	Duration, minutes per call/visit		
		Mean (SD)	Median (IQR)	Minimum-maximum
**Clinical**				
Phone calls	10	12.0 (6.4)	10 (9)	4-19
Home visits	5	24.0 (8.2)	30 (15)	15-30
**Technical**				
Phone calls	17	13.0 (10.3)	13 (10)	2-36
Home visits	15	49.0 (41.3)	35 (62.5)	5-123

IQR: interquartile range; SD: standard deviation

For peer review only

**Participant number and pseudonym**	**Quotation (page and line numbers)**
	**Training and Support**
P16 Ann (moderately impaired upper-limb)	*'He went through everything. He went through all the games as well, which was good, so that helped. So, it just gives you confidence and reassurance in what you're doing.' (7, 266-268).*
P22 Bal (moderately impaired upper-limb):	*'If I didn't understand or if the response wasn't what I was expecting then I would refer to the detailed instructions to see if I could improve (5, 192-94). Very easy [to use] […] The instructions were very clear (6, 229-237). [...] it's always useful to fall back on if you, if you can't cope.' (6, 247-48).*
P19 Sam (moderately impaired upper-limb):	*'Like I was stuck on a game, so I used the quick start just to find out if I was doing something wrong.' (13, 553-54).*
P28 Terry's wife Fran	*'I thought the quick start guide on that sheet was very helpful 'cos I'd often look at that while we were setting you up, weren't... didn't I, Terry, and that was very helpful, but then we didn't need it as time went on.'*
	**Acceptability**
P1 Iris (moderately impaired upper-limb)	*' I was trying to get to level 20 […] Yeah, I was getting into it […] I enjoyed the game because every time... If you didn't get to a, a certain point it will always say, 'Re-try' or 'Re-play' or 'Start again', and I like that (17, 777-94). And the message after was, was to tell you how many minutes you played for and 'Congratulations, see you tomorrow, bye', and that's it, I like that. [...] It was like you had a friend talking to you.' (21, 979-80).*
P6 Mark (mildly impaired upper-limb):	*'It was a little hard at first, right, because I used to be pressing it and pressing it but, er, anyth... anyway I got used to it like.' (21, 956-57).*
P16 Ann (moderately impaired upper-limb)	*'I like the ones that I can, that I actually can achieve, yeah. It's no good... I don't like ones that I know that I'm never gonna get to or never gonna do, I like ones that I can... Yeah, the easier ones for me that I can achieve relatively easy. Obviously it's gotta push you a little bit but not too much.' (14, 648-51)*

For peer review only - http://bmjopen.bmj.com/site/about/guidelines.xhtml

P22 Bal (moderately impaired upper-limb)	*'I have found it, er, quite entertaining but sometimes, because, er, there's not much variety, it's the same music, the same games, it's a bit boring to do it every day. [...] If there was a bit more variety or slightly different games, or even the games you could adjust so that, er, it was, er, slightly different, then I think it might not be so boring.' (15, 650-58).*

Box 1. Illustrative quotations from the qualitative interviews

For peer review only

For peer review only - http://bmjopen.bmj.com/site/about/guidelines.xhtml

Table 6 Change in outcomes between baseline and 8-weeks and baseline and 12-weeks.

Outcome	Baseline (n=30)	8-weeks (n=27)	Change; 8-weeks - baseline (n=27)	12-weeks (n=27)	Change; 12-weeks - baseline (n=27)
	Mean (SD)	Mean (SD)	Mean difference (95% CI); p value	Mean (SD)	Mean difference (95% CI); p value
ARAT[a] (0-57)	15.9 (17.5)	16.2 (17.3)	0.17 (-0.86 to 1.21); 0.743	18.2 (19.1)	0.63 (-0.97 to 2.23); 0.440
FMA-UE (0-66)	32.6 (16.4)	35.2 (17.6)	2.68 (0.86 to 4.49); 0.004	34.4 (17.8)	1.49 (-0.38 to 3.37); 0.118
SIPSO; total score (0-40)	21.9 (9.3)	23.5 (9.3)	1.18 (-0.81 to 3.18); 0.245	24.3 (8.1)	2.17 (0.32 to 4.01); 0.021
MAL (0-5)	0.89 (1.19)	1.08 (1.16)	0.19 (0.06 to 0.32); 0.004	1.10 (1.19)	0.13 (-0.02 to 0.28); 0.080
MMAS (0-5)	2.0 (1.0)	1.8 (1.0)	-0.16 (-0.38 to 0.06); 0.157	2.1 (1.1)	0.08 (-0.19 to 0.35); 0.560
EQ-5D-5L; utility score	0.49 (0.32)	0.56 (0.30)	0.07 (-0.009 to 0.142); 0.088	0.54 (0.32)	0.44 (-0.02 to 0.10); 0.145
Shoulder flexion[b]	121.3 (18.2)	123.8 (19.4)	3.09 (-2.17 to 8.37); 0.249	119.9 (21.2)	-0.26 (-7.31 to 6.77); 0.940
Shoulder external rotation[ab]	34.0 (19.9)	41.5 (16.7)	7.10 (2.36 to 11.84); 0.003	40.6 (16.4)	6.52 (0.02 to 13.03); 0.049
Shoulder abduction[b]	107.6 (17.0)	111.1 (19.3)	3.21 (-2.00 to 8.42); 0.228	108.7 (19.7)	1.48 (-4.66 to 7.63); 0.636
Elbow flexion[b]	142.3 (8.7)	145.9 (5.6)	3.28 (0.44 to 6.12); 0.024	148.4 (6.3)	6.00 (3.20 to 8.80); <0.001
Elbow extension[b]	-4.26 (10.84)	-4.14 (12.06)	0.54 (-1.94 to 3.03); 0.669	-4.29 (11.00)	0.39 (-1.79 to 2.58); 0.724
Wrist extension[b]	58.5 (29.1)	67.1 (19.6)	10.18 (1.31 to 19.05); 0.024	67.81 (23.22)	10.9 (1.8 to 20.1); 0.019

[a]n=26 at 12-weeks

For peer review only - http://bmjopen.bmj.com/site/about/guidelines.xhtml

[b] Passive range of movement, degrees

Discussion

The results of this feasibility study indicate that the Neurofenix is a safe, feasible and well accepted intervention for UL rehabilitation for participants more than one-year post-stroke with mild-to-severe arm impairment. Participants experienced a reduction in shoulder pain over time, no change in post-stroke fatigue, and mild short lived AEs. Participants were highly satisfied with the rehabilitation programme (see Box 1). Additionally, loss to follow-up and missing data were minimal, which in combination with data on safety, feasibility and acceptability, suggest that a definitive RCT is feasible.

On average, participants trained with the Neurofenix platform four days per week, for 149 minutes per week. Twenty-seven per cent achieved at least 225 minutes training per week which equates to the recommended 45 minutes of training per day for five days per week. [36] It may seem surprising, given the theoretical appeal of gamified therapy and its potential advantages over traditional home exercise programmes, that a larger proportion of participants were unable to achieve the recommended minimal dose. However, as community-based rehabilitation opportunities for stroke survivors typically reduce over time and rarely continue beyond six months [37]devices such as the Neurofenix platform offer a tangible means of increasing upper limb training hours from what is likely to be a very low base. [38] We do not have community based therapy comparison figures for other forms of UL therapy, but in the acute setting, where arguably patients have more access to therapists and UL treatment, only 7.9 minutes a day of combined occupational therapy and physiotherapy for the UL during the first few weeks post stroke has been reported.[39] Our participants also achieved a median (minimum-maximum) of 15,092 (222-43,999) UL movements over 7-weeks, which compares favourably to an average of 32 movement repetitions per session of inpatient upper limb rehabilitation reported by Lang et al.[40] So while adherence is variable in this study, problems related to persistence with technology-enhanced upper limb rehabilitation are consistent with previous studies using rehabilitation technology [41,42]. A recent systematic review concluded that further research is necessary to understand factors underlying perseverance with home-based technology-enhanced upper limb training post-stroke.[43] Standen at al [42] reported that family support, where available, was crucial to successful game-play. We also found that participants with severe UL impairment were dependent on carers for set-up.

For peer review only - http://bmjopen.bmj.com/site/about/guidelines.xhtml

Participants starting from a low baseline may apply an equivalent effort as those with less impairment without meeting training targets which suggests that personalised training goals may also be useful.

Our findings on safety are consistent with similar studies.[41,43,44] Self-limited use of the device permitted self-management of the level of training related muscle fatigue and may explain the improvement in pain. We systematically assessed AEs, regardless of their relatedness to the intervention, which may explain the relatively high proportion of participants who experienced at least one event. However, AEs were mild, and the majority were unrelated to the intervention, as found in comparable studies.[41,43]

Recruitment targets were achieved within the specified timeframe of five months; 60% of those screened were enrolled in the trial. The eligibility to enrolment ratio compares favourably to a multicentre study that examined the use of the Nintendo Wii for UL stroke rehabilitation in the UK where a low enrolment figure (4%) was achieved using broad eligibility screening in ten stroke centres.[41] Our strategy of using three recruitment streams, targeting local stroke groups and newsletters for stroke survivors, positively influenced engagement with the study and enrolment. However, other modes of recruitment may be necessary to enrol sufficient numbers for a definitive trial. Attrition was within acceptable limits (<20%),[45] and, compared to similar trials, consistent with figures reported by Adie et al [41] and lower than those reported by Standen et al.[42] Both studies provided comparable levels of support and participants were of similar age to those in our study. There are no obvious differences to account for the increased drop out in the Standen et al trial.[46]
Outcome data reporting was very good. Some participants found the assessments time consuming and fatiguing. We assessed the four core measures recommended for stroke trials.[46] We also included safety measures for pain and post-stroke fatigue, and the SIPSO because there is no consensus about the most appropriate measure of participation in stroke trials.[46] To balance the need to fully test our research questions against the need to use outcome measures judiciously [46] we additionally measured spasticity, passive range of movement and the MAL.

Most participants in this trial required one training session to confidently use the device and no further in-trial technical or clinical support. These results compare favourably with Standen et al [42] suggesting

For peer review only - http://bmjopen.bmj.com/site/about/guidelines.xhtml

the training and follow-up support required to implement this intervention is feasible in clinical practice. A minority of participants required phone calls and home visits to help with clinical and technical issues. These problems may impede fidelity and, as previously reported,[42] are potential barriers to independent home use of rehabilitation devices. The Neurofenix platform was designed to enable independent home-based rehabilitation. This study showed that although technology was effectively leveraged to reduce clinical contact time, individually paced home-based instruction and support services were necessary to effectively deliver the intervention. These additional hours would need to be factored into a fully scaled trial to ensure that technological interventions are not studied as a dose matched control but on how they would be rolled out and used by service users and clinicians in real life. A future RCT should examine the cost-effectiveness of the intervention compared to usual care, given the minimal therapist time involved in providing this intervention in a community setting.

Consistent with our findings, previous studies reported that participants generally enjoy using VR rehabilitation devices.[43,47,48] The compact size and light weight of the devices and the tablet facilitated the use of the platform in the home environment and positively influenced satisfaction and acceptability. Qualitative data suggested that a broader selection of games with a wide range of difficulty levels and the introduction of real time in-play competition and comparison may increase motivation and encourage persistence. These findings are consistent with game theory for stroke rehabilitation which emphasises the importance of in-built skill level adaptation, feedback, competition, and socialisation.[49] Adaptations to the games may be required prior to conducting a definitive trial to increase motivation and persistence.

To be maximally inclusive, stroke survivors with moderate to severe arm impairment were included in the study which may be considered a strength. Participants were relatively young compared to the UK average age of onset for stroke [50] but with a comparable age distribution to participants included in similar studies.[42,43,51] Older people may be thought to be less inclined to use rehabilitation technology, but we found no associations between age and time spent training using the device. The Neurofenix platform was custom designed for therapeutic use, which confers advantages over commercial gaming devices.[49] The device calibrates individual movement repetitions but key design features that further augment motivation and persistence such as personalisation through in-game adaptation [49,52] were not included in our device at the time of the study.

For peer review only - http://bmjopen.bmj.com/site/about/guidelines.xhtml

Study Limitations

There are methodological limitations to this study. The study design did not include a control group, which while this in in keeping with a feasibility study it did not allow us to test the willingness of participants to be randomised in a definitive RCT. Furthermore, the study used non-blinded assessors which may have generated higher effect estimates than blinded assessors.[53]Participants were relatively young compared to the UK average age of onset for stroke [50] but with a comparable age distribution to participants included in similar studies.[42,43,51]. Stroke survivors can experience pain from varied stroke related causes such as central post-stroke pain, pain from spasticity or contractures as well as from pre-existing co-morbidities; in addition some stroke survivors also had communication difficulties which made the measurement of pain challenging.[54]

Conclusion

The Neurofenix platform is a safe, feasible, and well-accepted means of delivering upper-limb rehabilitation to people following stroke. Iterative design continues to develop and improve the platform by incorporating game theory to encourage motivation and persistence. As the effects of stroke vary considerably across individuals, and as individuals are likely to have different technical capacities, a supported remote training model such as that used in this trial is likely to be needed in a definitive trial.

For peer review only

For peer review only - http://bmjopen.bmj.com/site/about/guidelines.xhtml

Author contributions

CK and GSB conceived the study, DS, TB, AN, JR, NA, AW, MN designed the study. GSB, DA, DS, TB, and CK designed the intervention. CK led the running of the trial. DS and TB led the collection, management, and analysis of the data. KB led blinded ARAT assessment. MN led the process evaluation. JR led the statistical analysis. NA led the evaluation of the feasibility of the economic evaluation. EC led the qualitative analysis. All authors have read and approved the final manuscript.

Study sponsor and funders were not involved in study design; collection; management; analysis and interpretation of the data; writing of the protocol; and the decision to submit the report for publication.

Acknowledgements

The authors would like to thank two stroke survivors who assisted the development of the intervention and advised on the protocol, trial documentation and dissemination. Further thanks to the group facilitators of Different Strokes and the Action for Rehabilitation from Neurological Injury. Thanks to Professor Christina Victor for her support.

Funding statement

This work was supported by Innovate UK grant number 104188, (the views expressed are those of the authors and not necessarily those of Innovate UK.)

Competing interests statement

GSB and DA are employed by Neurofenix (United Kingdom), a digital therapeutics company developing a therapy platform and sensor-based devices to augment rehabilitation. Neurofenix provided the Neurofenix platforms and technical support to the research therapists. Neurofenix had no influence on the design of the study, data collection, analysis and interpretation of the data or manuscript preparation. KB and DS were employed by Brunel University London during the period of the study, and are now Neurofenix employees.

All other authors declare no competing interests.

Patient consent

Informed written consent was obtained.

For peer review only

Ethics approval

Ethical approval was granted by the College of Health and Life Sciences Research Ethics Committee (REC) in Brunel University London (10249-MHR-Mar/2018-12322-2). Participants gave informed written consent before taking part.

Provenance and peer review

Not commissioned; externally peer reviewed.

Data statement

Participants did not consent for datasets to be stored or accessed outside of the research team. Therefore, no datasets have been made publicly available.

For peer review only

Reference List

1. Broeks JG, Lankhorst GJ, Rumping K, *et al.* The long-term outcome of arm function after stroke: results of a follow-up study. *Disabil Rehabil* 1999;21:357–64.

2. Nakayama H, Jørgensen HS, Raaschou HO, *et al*. Recovery of upper extremity function in stroke patients: the Copenhagen Stroke Study. *Arch Phys Med Rehabil* 1994;75(4):394-8.

3. Kwakkel G, Kollen BJ. Predicting activities after stroke: what is clinically relevant? *International J Stroke* 2013;8(1):25-32.

4. Lohse KR, Lang CE, Boyd LA. Is more better? Using Metadata to Explore Dose–Response Relationships in Stroke Rehabilitation. *Stroke* 2014;45:2053-2058.

5. Vratsistas-Curto A, Sherrington C, McCluskey A. Dosage and predictors of arm practice during inpatient stroke rehabilitation: an inception cohort study. *Disabil Rehabil* 2019;43(5):640-647.

6. Hayward KS, Brauer SG. Dose of arm activity training during acute and subacute rehabilitation post-stroke: A systematic review of the literature. *Clin Rehabil* 2015;29(12):1234-43.

7. Baniña MC, Molad R, Solomon JM, *et al.* Exercise intensity of the upper-limb can be enhanced using a virtual rehabilitation system. *Disabil Rehabil Assist Technol* 2020 DOI:10.1080/17483107.2020.1765421

8. Brunner I, Sture Skouen J, Hofstad H, *et al.* Is upper-limb virtual reality training more intensive than conventional training for patients in the subacute phase after stroke? An analysis of treatment intensity and content. *BMC Neurol* 2016;16:219. DOI 10.1186/s12883-016-0740-y.

9. Levac D, Glegg SM, Sveistrup H, *et al.* A knowledge translation intervention to enhance clinical application of a virtual reality system in stroke rehabilitation. *BMC Health Serv Res* 2016;16(1):557.

10. Wingham J, Adie K, Turner D, *et al.* Participant and caregiver experience of the Nintendo Wii SportsTM after stroke: qualitative study of the trial of WiiTM in stroke (TWIST). *Clin Rehabil* 2015;29(3):295-305.

11. Tsekleves E, Paraskevopoulos IT, Warland A, *et al.* Development and preliminary evaluation of a novel low cost VR-based upper-limb stroke rehabilitation platform using Wii technology. *Disabil Rehabil Assist Technol* 2016;11(5):413-22.

For peer review only - http://bmjopen.bmj.com/site/about/guidelines.xhtml

12. Eldridge SM, Chan CL, Campbell MJ, *et al.* CONSORT 2010 statement: extension to randomised pilot and feasibility trials. *BMJ* 2016; 355:i5239.

13. Kilbride C, Scott DJ, Butcher T, *et al.* Rehabilitation via HOMe Based gaming exercise for the Upper-limb post-stroke (RHOMBUS): protocol of an intervention feasibility trial. *BMJ Open*. 2018;8(11):e026620.

14. Bruno A, Akinwuntan AE, Lin C, *et al.* Simplified modified Rankin scale questionnaire: reproducibility over the telephone and validation with quality of life. *Stroke* 2011;42(8):2276-9.

15. Bruno A, Shah N, Akinwuntan AE, *et al.* Stroke size correlates with functional outcome on the simplified modified Rankin Scale questionnaire. *J Stroke Cerebrovasc Dis* 2013;22(6):781-3.

16. Sivan M, O'Connor RJ, Makower S, *et al.* Systematic review of outcome measures used in the evaluation of robot-assisted upper-limb exercise in stroke. *J Rehabil Med* 2011;43(3):181-9.

17. Van der Lee, JH, De Groot V, Beckerman H, *et al.* The intra-and interrater reliability of the action research arm test: a practical test of upper extremity function in patients with stroke. *Arch Phys Med Rehabil* 2001;82(1):14-9.

18. Yozbatiran N, Der-Yeghiaian L, Cramer SC. A standardized approach to performing the action research arm test. *Neurorehabil Neural Repair* 2008 Jan-Feb;22(1):78-90.

19. Page SJ, Fulk GD, Boyne P. Clinically important differences for the upper-extremity Fugl-Meyer Scale in people with minimal to moderate impairment due to chronic stroke. *Phys Ther* 2012;92(6):791-8.

20. Duncan PW, Propst M, Nelson SG. Reliability of the Fugl-Meyer assessment of sensorimotor recovery following cerebrovascular accident. *Phys Ther* 1983;63(10):1606-10.

21. Trigg R, Wood VA. The Subjective Index of Physical and Social Outcome (SIPSO): a new measure for use with stroke patients. *Clin Rehabil* 2000;14(3):288-99.

22. Simpson LA, Eng JJ. Functional recovery following stroke: capturing changes in upper-extremity function. *Neurorehabil Neural Repair* 2013;27(3):240-50.

23. Van der Lee, JH, Beckerman H, Knol DL, *et al.* Clinimetric properties of the motor activity log for the assessment of arm use in hemiparetic patients. *Stroke* 2004;35(6):1410-4.

For peer review only - http://bmjopen.bmj.com/site/about/guidelines.xhtml

24. Bohannon RW, Smith MB. Interrater reliability of a modified Ashworth scale of muscle spasticity. *Phys Ther* 1987;67(2):206-7.

25. van de Pol, RJ, van Trijffel E, Lucas C. Inter-rater reliability for measurement of passive physiological range of motion of upper extremity joints is better if instruments are used: a systematic review. *J Physiother* 2010;56(1):7-17.

26. Herdman M, Gudex C, Lloyd A, *et al.* Development and preliminary testing of the new five-level version of EQ-5D (EQ-5D-5L). *Qual Life Res* 2011;20(10):1727-36.

27. van Hout B, Janssen MF, Feng YS, *et al.* Interim scoring for the EQ-5D-5L: mapping the EQ-5D-5L to EQ-5D-3L value sets. *Value Health* 2012;15:708-15.

28. Rodgers H, Shaw L, Cant R, *et al.* Evaluating an extended rehabilitation service for stroke patients (EXTRAS): study protocol for a randomised controlled trial. *Trials* 2015;16(1):205.

29. Beecham J, Knapp M. Costing psychiatric interventions. *Measuring Mental Health Needs.* 2001;2:200-24.

30. Lerdal A, Kottorp A. Psychometric properties of the Fatigue Severity Scale—Rasch analyses of individual responses in a Norwegian stroke cohort. *Int J Nurs Stud* 2011;48(10):1258-65.

31. Demers L, Weiss-Lambrou R, Ska B. The Quebec user evaluation of satisfaction with assistive technology (QUEST 2.0): an overview and recent progress. *Technol Disabil* 2002;14:101-105.

32. Sim J, Lewis M. The size of a pilot study for a clinical trial should be calculated in relation to considerations of precision and efficiency. *J Clin Epidemiol* 2012;65:301-308.

33. SA Julious, NE McIntyre. Sample size of 12 per group rule of thumb for a pilot study. *Pharm Stat* 2005;4: 287-291

34. Gale NK, Heath G, Cameron E, et al. (2013) Using the framework method for the analysis of qualitative research data in multi-disciplinary health research. *BMC Med Res Methodol,* 2013;3,117 hyyp://www.biomedcentral.com/1471-2288/13/117.

35. Holloway I, Galvin K. *Qualitative research in nursing and healthcare.* John Wiley & Sons; 2016.

36. Intercollegiate Stroke Working Party. *National Clinical Guidelines for Stroke.* 5th edn. London: Royal College of Physicians. 2016

For peer review only

For peer review only - http://bmjopen.bmj.com/site/about/guidelines.xhtml

https://www.strokeaudit.org/SupportFiles/Documents/Guidelines/2016-National-Clinical-Guideline-for-Stroke-5t-(1).aspx

37. Subramanian, S., Massie, C., Malcolm, M. and Levin, M. Does provision of extrinsic feedback result in improved motor learning in the upper limb post-stroke? A systematic review of the evidence. *Neurorehabil Neural Repair* 2010; 24(2):113-124.

38. Ward, N. Restoring brain function after stroke-bridging the gap between animals and humans. *Nat Rev Neurol* 2017; 13(4): 244-255.

39. Serrada I, McDonnell MN, Hillier SL. What is current practice for upper limb rehabilitation in the acute hospital setting following stroke? A systematic review. *NeuroRehabilitation* 2016; 15;39(3):431-8. doi: 10.3233/NRE-161374. PMID: 27589513.

40. Lang C, Macdonald J, Reisman D, *et al.* Observation of amounts of movement practice provided during stroke rehabilitation. *Arch Phys Med Rehab* 2009; 90(10): 1692-1698.

41. Adie K, Schofield C, Berrow M, *et al.* Does the use of Nintendo Wii SportsTM improve arm function? Trial of WiiTM in Stroke: a randomized controlled trial and economics analysis. *Clin Rehabil* 2017;31(2) 173–185. DOI: 10.1177/0269215516637893

42. Standen PJ, Threapleton K, Connell L, *et al.* Patients' use of a home-based virtual reality system to provide rehabilitation of the upper-limb following stroke. *Phys Ther* 2015;95:350-359.

43. Laver KE, Lange B, George S, *et al.* Virtual reality for stroke rehabilitation. *Cochrane Database Syst Rev* 2017, Issue 11. Art. No.: CD008349. DOI: 10.1002/14651858.CD008349.pub4.

44. Chen Y, Abel KT, Janecek JT, *et al.* Home-based technologies for stroke rehabilitation: A systematic review. *Int J Med Inform* 2019;123:11-22.

45. Schultz KF, Grimes DA. Sample size slippages in randomised controlled trials: exclusions, the lost and the wayward. *Lancet* 2002;359:781-85.

46. Kwakkel G, Lannin NA, Borschmann K, *et al.* Standardized measurement of sensorimotor recovery in stroke trials: consensus-based core recommendations from the Stroke Recovery and Rehabilitation Roundtable. *Neurorehabil Neural Repair* 2017;31(9):784–792.

For peer review only

47. Syed UE, Kamal A. Video game-based and conventional therapies in patients of neurological deficits: an experimental study. *Disabil Rehabil Assist Technol* 2019;DOI: 10.1080/17483107.2019.1679266.

48. Lehmann I, Baer G, Schuster-Amft C. Experience of an upper-limb training program with a non-immersive virtual reality system in patients after stroke: a qualitative study. *Physiother* 2020;107:317-326.

49. Barrett N, Swain I, Gatzidis C, *et al.* The use and effect of video game design theory in the creation of game-based systems for upper-limb stroke rehabilitation. *J Rehabil Assist Technol Eng* 2016;3:1-16.

50. National Institute for Health and Care Excellence Guideline. Stroke and transient ischaemic attack in over 16s: diagnosis and initial management. NICE Guideline [NG128]:2019 https://www.nice.org.uk/guidance/ng128.

51. Domínguez-Téllez P, Moral-Muñoz JA, Salazar A, *et al.* Game-based virtual reality interventions to improve upper-limb motor function and quality of life after stroke: systematic review and meta-analysis. *Games Health J* 2020;9:1-10.

52. Pezzera M, Borghese NA. Dynamic difficulty adjustment in exer-games for rehabilitation: a mixed approach. *IEEE 8th International Conference on Serious Games and Applications for Health (SeGAH),* 2020; DOI 10.1109/SeGAH49190.2020.9201871.

53. Hrobjartsson A, Thomsen ASS, Emanuelsson F, *et al.* Observer bias in randomised clinical trials with binary outcomes: systematic review of trials with both blinded and non-blinded outcome assessors. *BMJ* 2012; 344 :e1119 doi:10.1136/bmj.e119

54. Nesbitt J, Moxham S, Ramaduraj G. *et al.* Improving pain assessment and management in stroke patients. *BMJ Qual Improv Rep.* 2015; 4(1), doi:10.1136/bmjquality.u203375.w3105

For peer review only

For peer review only - http://bmjopen.bmj.com/site/about/guidelines.xhtml

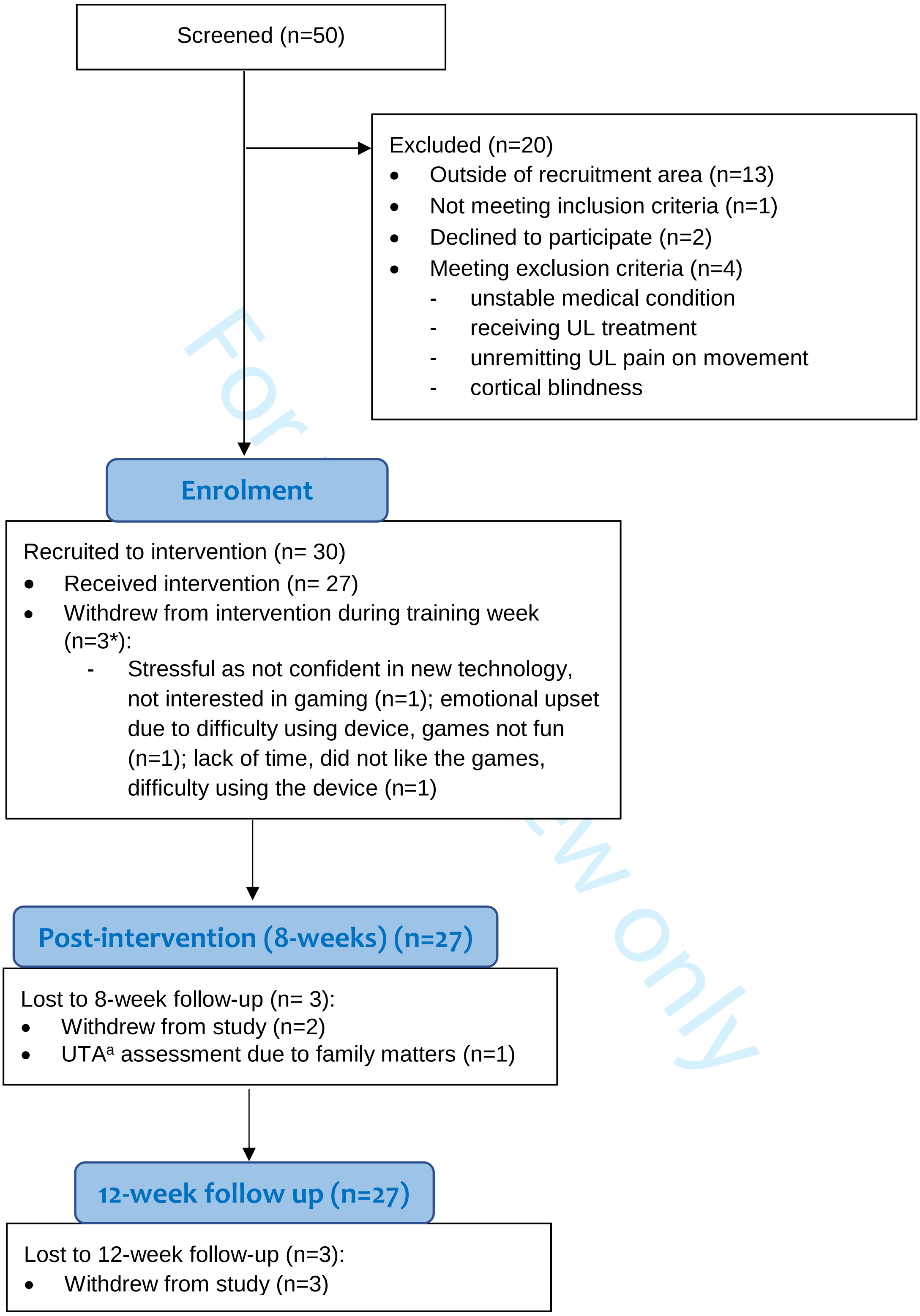

Figure 1. CONSORT flow of participants through the trial.
[a]Unable to attend.

For peer review only

CONSORT 2010 checklist of information to include when reporting a pilot or feasibility trial*

Section/Topic	Item No	Checklist item	Reported on page No
**Title and abstract**			
	1a	Identification as a pilot or feasibility randomised trial in the title	1
	1b	Structured summary of pilot trial design, methods, results, and conclusions (for specific guidance see CONSORT abstract extension for pilot trials)	2,3
**Introduction**			
Background and objectives	2a	Scientific background and explanation of rationale for future definitive trial, and reasons for randomised pilot trial	5
	2b	Specific objectives or research questions for pilot trial	5
**Methods**			
Trial design	3a	Description of pilot trial design (such as parallel, factorial) including allocation ratio	6
	3b	Important changes to methods after pilot trial commencement (such as eligibility criteria), with reasons	n/a
Participants	4a	Eligibility criteria for participants	6
	4b	Settings and locations where the data were collected	6
	4c	How participants were identified and consented	6
Interventions	5	The interventions for each group with sufficient details to allow replication, including how and when they were actually administered	6,7
Outcomes	6a	Completely defined prespecified assessments or measurements to address each pilot trial objective specified in 2b, including how and when they were assessed	7,8
	6b	Any changes to pilot trial assessments or measurements after the pilot trial commenced, with reasons	n/a
	6c	If applicable, prespecified criteria used to judge whether, or how, to proceed with future definitive trial	n/a
Sample size	7a	Rationale for numbers in the pilot trial	9
	7b	When applicable, explanation of any interim analyses and stopping guidelines	n/a
Randomisation:			
Sequence generation	8a	Method used to generate the random allocation sequence	n/a
	8b	Type of randomisation(s); details of any restriction (such as blocking and block size)	n/a
Allocation concealment mechanism	9	Mechanism used to implement the random allocation sequence (such as sequentially numbered containers), describing any steps taken to conceal the sequence until interventions were assigned	n/a

For peer review only

Implementation	10	Who generated the random allocation sequence, who enrolled participants, and who assigned participants to interventions	n/a
Blinding	11a	If done, who was blinded after assignment to interventions (for example, participants, care providers, those assessing outcomes) and how	n/a
	11b	If relevant, description of the similarity of interventions	n/a
Statistical methods	12	Methods used to address each pilot trial objective whether qualitative or quantitative	9,10
**Results**			
Participant flow (a diagram is strongly recommended)	13a	For each group, the numbers of participants who were approached and/or assessed for eligibility, randomly assigned, received intended treatment, and were assessed for each objective	11, Fig 1
	13b	For each group, losses and exclusions after randomisation, together with reasons	11, Fig 1
Recruitment	14a	Dates defining the periods of recruitment and follow-up	10
	14b	Why the pilot trial ended or was stopped	n/a
Baseline data	15	A table showing baseline demographic and clinical characteristics for each group	11
Numbers analysed	16	For each objective, number of participants (denominator) included in each analysis. If relevant, these numbers should be by randomised group	12, 13
Outcomes and estimation	17	For each objective, results including expressions of uncertainty (such as 95% confidence interval) for any estimates. If relevant, these results should be by randomised group	19
Ancillary analyses	18	Results of any other analyses performed that could be used to inform the future definitive trial	13
Harms	19	All important harms or unintended effects in each group (for specific guidance see CONSORT for harms)	14,17
	19a	If relevant, other important unintended consequences	n/a
**Discussion**			
Limitations	20	Pilot trial limitations, addressing sources of potential bias and remaining uncertainty about feasibility	4, 20-22,
Generalisability	21	Generalisability (applicability) of pilot trial methods and findings to future definitive trial and other studies	22
Interpretation	22	Interpretation consistent with pilot trial objectives and findings, balancing potential benefits and harms, and considering other relevant evidence	20-22,
	22a	Implications for progression from pilot to future definitive trial, including any proposed amendments	22
**Other information**			
Registration	23	Registration number for pilot trial and name of trial registry	3
Protocol	24	Where the pilot trial protocol can be accessed, if available	5
Funding	25	Sources of funding and other support (such as supply of drugs), role of funders	23
	26	Ethical approval or approval by research review committee, confirmed with reference number	24

For peer review only

Citation: Eldridge SM, Chan CL, Campbell MJ, Bond CM, Hopewell S, Thabane L, et al. CONSORT 2010 statement: extension to randomised pilot and feasibility trials. BMJ. 2016;355.
*We strongly recommend reading this statement in conjunction with the CONSORT 2010, extension to randomised pilot and feasibility trials, Explanation and Elaboration for important clarifications on all the items. If relevant, we also recommend reading CONSORT extensions for cluster randomised trials, non-inferiority and equivalence trials, non-pharmacological treatments, herbal interventions, and pragmatic trials. Additional extensions are forthcoming: for those and for up to date references relevant to this checklist, see www.consort-statement.org.

For peer review only
